# Extending the time window for tenecteplase by effective reperfusion of penumbral tissue in patients with large vessel occlusion: Rationale and design of a multicenter, prospective, randomized, open-label, blinded-endpoint, controlled phase 3 trial

**DOI:** 10.1177/17474930241308660

**Published:** 2024-12-31

**Authors:** Vignan Yogendrakumar, Bruce CV Campbell, Leonid Churilov, Carlos Garcia-Esperon, Philip MC Choi, Dennis J Cordato, Prodipta Guha, Gagan Sharma, Chushuang Chen, Amy McDonald, Vincent Thijs, Abul Mamun, Angela Dos Santos, Anna H Balabanski, Timothy J Kleinig, Ken S Butcher, Michael J Devlin, Fintan O’Rourke, Geoffrey A Donnan, Stephen M Davis, Christopher R Levi, Henry Ma, Mark W Parsons

**Affiliations:** 1 Department of Neurology, Melbourne Brain Centre, The Royal Melbourne Hospital, The University of Melbourne, Parkville, VIC, Australia; 2 Department of Medicine, The University of Melbourne, Parkville, VIC, Australia; 3 Division of Neurology, Department of Medicine, The Ottawa Hospital, Ottawa Hospital Research Institute, University of Ottawa, Ottawa, ON, Canada; 4 Hunter New England Local Health District, New Lambton Heights, NSW, Australia; 5 Faculty of Medicine, University of Newcastle, Newcastle, NSW, Australia; 6 Department of Neuroscience, Box Hill Hospital, Eastern Health, Melbourne, VIC, Australia; 7 Department of Neurology, Liverpool Hospital, University of New South Wales, Ingham Institute, Liverpool, NSW, Australia; 8 Florey Institute of Neuroscience and Mental Health, The University of Melbourne, Parkville, VIC, Australia; 9 Department of Medicine, Austin Health, The University of Melbourne, Heidelberg, VIC, Australia; 10 Department of Neurology, Campbelltown Hospital, Campbelltown, NSW, Australia; 11 School of Clinical Medicine, University of New South Wales, Sydney, NSW, Australia; 12 Department of Neuroscience, School of Translational Medicine, Alfred Health, Monash University, Melbourne, VIC, Australia; 13 Department of Neurology, Royal Adelaide Hospital, Adelaide, SA, Australia; 14 Department of Neurology, Princess Alexandra Hospital, Brisbane, QLD, Australia; 15 Department of Aged Care, Stroke and Rehabilitation, Bankstown-Lidcombe Hospital, University of New South Wales, Sydney, NSW, Australia; 16 School of Clinical Science at Monash Health, Department of Medicine and Neurology, Monash University, Melbourne, VIC, Australia

**Keywords:** Acute stroke therapy, ischemic stroke, treatment, tPA, thrombolysis, TNK

## Abstract

**Rationale:**

The benefit of tenecteplase in the treatment of large vessel occlusion (LVO) patients presenting within 24 h of symptom onset remains unclear.

**Aim:**

This study aimed to assess the effectiveness and safety of tenecteplase, compared to standard of care, in patients presenting within the first 24 h of symptom onset with an LVO and target mismatch on perfusion computed tomography (CT).

**Methods and design:**

The “Extending the time window for Tenecteplase by Effective Reperfusion of peNumbrAL tissue in patients with Large Vessel Occlusion” (ETERNAL-LVO) trial is a prospective, randomized, open-label, blinded-endpoint, phase 3, parallel-group, superiority trial with covariate-adjusted 1:1 randomization, and adaptive sample size re-estimation. Patients with an anterior circulation LVO stroke, who present within 24 h of stroke onset or last known well with a target mismatch on computed tomography perfusion (CTP) or magnetic resonance imaging (MRI), will be randomized to tenecteplase (0.25 mg/kg) or standard of care (alteplase 0.90 mg/kg or conservative management at clinician discretion) prior to undergoing endovascular therapy.

**Study outcomes:**

The primary outcome is the proportion of patients with a modified Rankin Scale (mRS) of 0–1 (no disability) or return to baseline mRS at 3 months. Secondary and safety outcomes include the proportion of patients with an mRS of 0–2 at 3 months, an ordinal analysis of the mRS at 3 months, the proportion of patients with symptomatic intracerebral hemorrhage (sICH), the proportion of patients with death due to any cause, and the proportion of patients with mRS 5–6 at 3 months (severe disability or death).

**Discussion:**

The ETERNAL-LVO trial will build on the current evidence for tenecteplase in the > 4.5-h window. Specifically, this trial will evaluate tenecteplase in a patient population who have access to endovascular therapy but may incur delays to endovascular therapy commencement or require transfer from a primary to a comprehensive stroke center.

**Trials registration:**

ClincialTrials.gov: NCT04454788.

## Introduction and rationale

In the management of acute ischemic stroke, alteplase, the sole treatment available since the 1990s, is being replaced by tenecteplase, a closely related variant that can be administered in a single bolus dose and whose pharmacological properties allow for potentially improved thrombus clearance. The use of tenecteplase within the 4.5-h treatment window is supported by three non-inferiority randomized controlled trials (RCTs)^1–3^ and several phase 2 studies.^4,5^

However, the evidence supporting the use of tenecteplase in the treatment of patients presenting beyond 4.5 h, specifically those with a large vessel occlusion (LVO), remains unclear. The TIMELESS trial, comparing tenecteplase with placebo in LVO patients presenting 4.5–24 h after symptom onset, did not show a significant benefit in long-term outcome with tenecteplase use.^6^ In TIMELESS, 96% of patients were recruited at comprehensive stroke centers and the majority underwent endovascular therapy. The median time from tenecteplase administration to arterial puncture was 15 min, allowing very little time for thrombolytic effect. In contrast, the TRACE-III trial compared tenecteplase with standard of care in an LVO cohort who presented beyond 4.5 h and did not have timely access to endovascular therapy.^7^ In this context, tenecteplase was found to improve patient outcomes at 3 months.

The results of TIMELESS and TRACE-III are relevant to two unique patient populations: those with rapid access to endovascular therapy and those without access. However, many patients worldwide have access to endovascular therapy through an interhospital transfer from a primary stroke center where a thrombolytic can be administered, to a comprehensive stroke center where endovascular therapy can be performed. These patients, in whom the effectiveness of tenecteplase remains unknown, were among those included in the ETERNAL-LVO RCT.

## Methods

### Design

ETERNAL-LVO is a prospective, randomized, open-label, blinded-endpoint (PROBE), phase 3, parallel-group, superiority trial with covariate-adjusted 1:1 randomization, and adaptive sample size re-estimation. The study protocol is provided as Supplemental Material, and the trial is registered at Clinicaltrials.gov (NCT04454788). The study was approved by the University of Melbourne Ethics Committee (UOM2102). Written informed consent was obtained from the participant or a legal representative before enrollment, except in jurisdictions where deferral of consent for emergency treatment was allowed, in which case consent was obtained at a later time point to continue participation.

### Patient population

Adult patients (⩾ 18 years of age) presenting with an anterior circulation LVO ischemic stroke, who are eligible for standard intravenous therapy, and who present within 24 h of stroke onset or last known well. In addition to standard thrombolysis eligibility criteria, the presence of salvageable tissue without a large ischemic core on perfusion computed tomography (CTP) or magnetic resonance imaging (MRI) is an additional requirement for trial enrollment. Key inclusion and exclusion criteria are provided below (see protocol for full list).

#### Inclusion criteria

- Patients presenting with acute hemispheric ischemic stroke with onset (or the time they last known to be well) within 24 h.
- Patient’s age is ⩾ 18 years.
- Premorbid modified Rankin Scale (mRS) < 3 with ability, immediately prior to the stroke, to: (1) Drive, or (if never drove) perform own domestic duties, (2) shop for themselves, and (3) bank/manage their own finances.
- Presence of an anterior circulation LVO on CT angiography (CTA) or magnetic resonance angiography (MRA). LVO will be defined as “potentially retrievable” thrombus at one or more of the following sites: extracranial or intracranial internal carotid (ICA), middle cerebral artery (MCA) first segment (M1) and proximal MCA second segment (M2).
- Presence of “target mismatch” on CTP or diffusion–perfusion MRI as processed by MIStar or RAPID software. Mismatch is defined as an ischemic core of < 70 mL, penumbra of > 15 mL, and a perfusion-to-ischemic core lesion ratio > 1.8.

#### Exclusion criteria

- Intracranial hemorrhage (ICH) or other diagnosis (e.g. tumor).
- Extensive early ischemic change (hypodensity on non-contrast CT [NCCT] or high signal on diffusion-weighted imaging (DWI)-MRI) or early ischemic change outside the perfusion lesion that invalidates mismatch criteria.
- Pre-stroke mRS score of > 2 (indicating significant previous disability).
- Any terminal illness such that patient would not be expected to survive more than 1 year.
- Any condition that, in the judgment of the investigator, could impose hazards to the patient if study therapy is initiated or affect the participation of the patient in the study.

For patients taking anticoagulants, if a patient is on warfarin, enrollment is possible if the point of care INR ⩽ 1.4 or laboratory INR ⩽ 1.7. If dabigatran is known or suspected to have been taken within the last 48 h, then Idarucizumab 5 g IV bolus can be given prior to enrollment. Finally, if apixaban/rivaroxaban is known to have been taken in the last 12 h, then the patient cannot be enrolled.

### Randomization

Randomization will be computer-generated, using a covariate-adjusted randomization procedure to first stratify on clinician intention to give alteplase or no thrombolysis if randomized to standard of care and then minimize imbalances of the following covariates within the above strata: age, National Institute of Health Stroke Scale (NIHSS), premorbid mRS, site of LVO (Intracranial ICA and M1 vs extracranial ICA and M2, with tandem ICA occlusions to be considered intracranial for the purpose of covariate adjustment), and onset-to-randomization time (0–4.5, 4.5–12 and 12–24 h).

### Treatment

Patients will receive open-label intravenous tenecteplase at a dose of 0.25 mg/kg (Metalyse; Boehringer Ingelheim, Germany), given as a bolus or “standard of care,” which will include intravenous alteplase at the standard dose of 0.9 mg/kg up to a maximum of 90 mg (10% as bolus and the remainder over 1 h, Actilyse; Boehringer Ingelheim, Germany), or no thrombolytic at the discretion of the treating clinician. Following medication administration, patients may undergo endovascular therapy if clinically indicated.

### Primary outcomes

**Figure 1.**
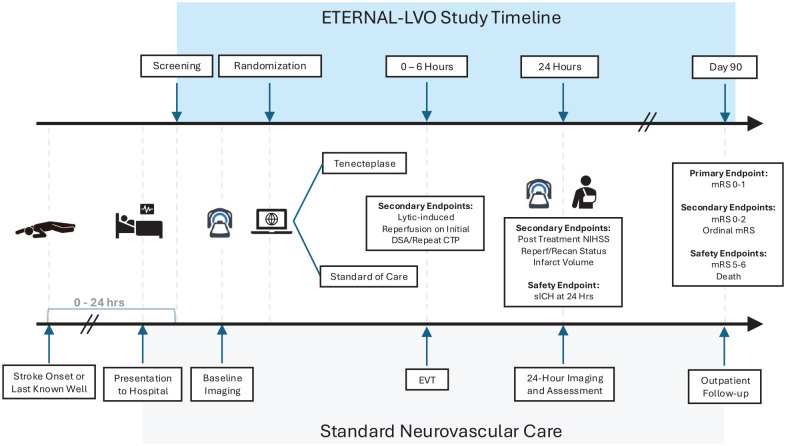
Trial flow diagram. DSA: digital subtraction angiography; EVT: endovascular therapy; CTP: CT perfusion; NIHSS: National Institute of Health Stroke Scale; reperf: reperfusion; recan: recanalization; sICH: symptomatic intracranial hemorrhage; mRS: modified Rankin Scale.

The proportion of patients with an mRS of 0–1 (no disability) or return to baseline mRS, if premorbid mRS was 2, at 3 months (Figure 1).

### Secondary outcomes

Secondary outcomes include the proportion of patients with mRS 0–2 at 3 months, an ordinal analysis of the mRS at 3 months, the proportion of patients with early thrombolytic-induced reperfusion, defined as the absence of retrievable thrombus and > 50% perfusion in the vascular bed of interest (e.g. TICI 2b/3) on initial digital subtraction angiography run, and the proportion of patients achieving early clinical improvement (defined as a reduction in the 24-h NIHSS score of ⩾ 8 points or a 24-h NIHSS of 0–1). Safety outcomes include the proportion of patients with symptomatic intracerebral hemorrhage (sICH), the proportion of patients with death due to any cause, and the proportion of patients with mRS 5–6 at 3 months (severe disability or death). sICH is defined as parenchymal hematoma type 2 (PH2—blood clot occupying > 30% of the infarcted territory with substantial mass effect) within 36 h of treatment combined with neurological deterioration leading to an increase in ⩾ 4 points on the NIHSS from baseline, or the lowest NIHSS value between baseline and 24 h. Exploratory outcomes will include the proportion of patients achieving reperfusion (> 90%) at 24-h post-stroke, infarct growth at 24 h, and the proportion of patients achieving successful recanalization at 24-h post-stroke.

### Data monitoring body

The trial DSMB comprises two independent clinical thrombolysis trial experts and one statistician to ensure ethical conduct of the trial and protect the rights and welfare of participants. The trial DSMB will receive six monthly reports and meet as needed.

### Sample size estimates

An estimated total sample size of 740 patients, equally distributed between two study arms, will yield 80% power to detect an absolute difference of at least 10% in the proportion of patients achieving the primary outcome between two arms using a two-sided statistical significance threshold of p = 0.05. The estimates in the “standard care” group are based on the outcomes of the EXTEND trial, where the proportions of LVO patients with the primary outcome (3-month mRS 0–1) were: placebo arm 25%, alteplase arm 28%, with the more conservative assumed proportion taken as 28%.^8^ The treatment effect of tenecteplase was based on the pooled analysis of phase 2 tenecteplase LVO data, where the tenecteplase treatment effect size was 19% greater than alteplase. A more conservative treatment effect of 10% has been assumed for ETERNAL. An adaptive increase in sample size is planned if the results of interim analysis using data from the first 592 patients are promising, as per the methodology of Mehta and Pocock.^9^ The maximum sample size is capped at 1000 patients (500 per arm).

### Statistical analysis

The analysis will be conducted following an intention-to-treat approach. Differences in all endpoints between the two arms of the study will be tested independently at the two-tailed 0.05 level of significance. All estimates of treatment effects will be presented with 95% confidence intervals. No formal adjustments will be undertaken to constrain the overall type I error associated with the secondary and exploratory analyses. Their purpose is to supplement evidence from the primary analysis to more fully characterize the treatment effect. Results from the secondary analyses will be interpreted in this context. Descriptive statistics will be generated for each of the measures used in the study.

The primary outcome will be analyzed using modified Poisson regression with mRS 0–1 (no disability) or return to premorbid mRS at 3 months as the dependent variable, treatment arm as an independent variable, and age, baseline NIHSS score, and onset-to-randomization time (categorized as 0–4.5 vs 4.5–12 vs 12–24 h) as treatment covariates. Treatment effect will be presented as adjusted risk ratio with corresponding 95% confidence interval (95%CI). The analyses of dichotomous secondary efficacy and safety outcomes will be conducted using the above approach, with each respective outcome as the dependent variable.

For the ordinal analysis of the Day 90 mRS, an ordinal logistic regression analysis model or an assumption-free generalized odds ratio (OR) model analysis will be undertaken on the full range (0–6) of the scale depending on whether the proportional odds assumption is satisfied. The details of the statistical analysis will be summarized in a separate Statistical Analysis Plan prior to the lock of the trial data.

### Study organization

Recruitment is planned at trial sites in Australia. The trial sponsor is the University of Melbourne, Melbourne, Australia. The first patient was enrolled on 1 August 2020.

### Funding

The trial is supported by a clinical trial and cohort grant from the National Health and Medical Research Council (ID: APP1182533), and also receives support from Boehringer Ingelheim.

## Discussion

The ETERNAL-LVO trial will build on the current evidence for tenecteplase in the > 4.5-h window. Specifically, by including both comprehensive and primary stroke centers as recruiting sites, this trial will evaluate tenecteplase in a patient population who have access to endovascular therapy but may incur delays to endovascular therapy or require transfer from a primary to a comprehensive stroke center. Past studies have demonstrated that administering a thrombolytic during this time delay can both increase the chances of early clot dissolution and reperfusion, and influence long-term clinical outcomes.^10,11^ Given the ongoing issues of endovascular therapy access being limited to urban centers and the common use of drip-and-ship models, the results of ETERNAL-LVO will be highly generalizable to many sites worldwide.

ETERNAL-LVO shares many common features with previously published trials evaluating tenecteplase in the late window, such as the use of perfusion imaging for patient selection. However, a distinct feature will be the inclusion of patients in the 0- to 4.5-h time window into the trial. The assessment of tenecteplase in this time window has been extensively explored in broad patient populations through several RCTs.^12^ Some may argue that this has been sufficiently assessed in the LVO population. While certain phase 2 trials of LVO patients have indeed shown benefit of tenecteplase over alteplase in the 0- to 4.5-h window,^5^ these findings have not been consistently observed in larger phase 3 subgroup analyses.^3,13^ As such, further evidence is required and ETERNAL-LVO will be well positioned to definitively assess tenecteplase in LVO patients in both the early (0–4.5 h) and delayed (4.5–24 h) time window.

Given the inclusion of both early and late time windows, in addition to the publication of studies, such as the EXTEND trial,^8^ we anticipate there will be some variation in the standard of care for patients. Specifically, we expect that some clinicians will now administer alteplase in the 4.5- to 9-h time window and that “standard of care” for LVO patients beyond 4.5 h could include IV alteplase or no thrombolytic. To accommodate this expected variation in practice, the “standard of care arm” will allow either 0.9 mg/kg alteplase or no lytic at the local investigator’s discretion, but with the control group treatment prespecified by the investigator (i.e. alteplase or not) prior to randomization. The use of covariate-adjusted randomization will minimize imbalances in key prognostic covariates between treatment groups.

## Summary and conclusion

The ETERNAL-LVO trial will compare tenecteplase to standard of care in patients presenting with an LVO and salvageable brain tissue within the first 24 h of symptom onset. This phase 3 trial will provide definitive evidence for tenecteplase in the early and delayed time windows and based on site selection for the study, it is highly generalizable to drip-and-ship patients and those with delayed access to endovascular therapy.

## Supplemental Material

sj-pdf-1-wso-10.1177_17474930241308660 – Supplemental material for Extending the time window for tenecteplase by effective reperfusion of penumbral tissue in patients with large vessel occlusion: Rationale and design of a multicenter, prospective, randomized, open-label, blinded-endpoint, controlled phase 3 trial Supplemental material, sj-pdf-1-wso-10.1177_17474930241308660 for Extending the time window for tenecteplase by effective reperfusion of penumbral tissue in patients with large vessel occlusion: Rationale and design of a multicenter, prospective, randomized, open-label, blinded-endpoint, controlled phase 3 trial by Vignan Yogendrakumar, Bruce CV Campbell, Leonid Churilov, Carlos Garcia-Esperon, Philip MC Choi, Dennis J Cordato, Prodipta Guha, Gagan Sharma, Chushuang Chen, Amy McDonald, Vincent Thijs, Abul Mamun, Angela Dos Santos, Anna H Balabanski, Timothy J Kleinig, Ken S Butcher, Michael J Devlin, Fintan O’Rourke, Geoffrey A Donnan, Stephen M Davis, Christopher R Levi, Henry Ma and Mark W Parsons in International Journal of Stroke
