# Supplementary material for: Extending the time window for tenecteplase by effective reperfusion of penumbral tissue in patients with large vessel occlusion: Rationale and design of a multicenter, prospective, randomized, open-label, blinded-endpoint, controlled phase 3 trial
Source: Int J Stroke. 2024 Dec 31;20(3):367–72. doi: 10.1177/17474930241308660 (PMC11874470; doi:10.1177/17474930241308660)
Supplement: sj-pdf-1-wso-10.1177_17474930241308660 – Supplemental material for Extending the time window for tenecteplase by effective reperfusion of penumbral tissue in patients with large vessel occlusion: Rationale and design of a multicenter, prospective, randomized, open-label, blinded-endpoint, controlled [file sj-pdf-1-wso-10.1177_17474930241308660.pdf]

CONFIDENTIAL

Protocol Title

Extending the time window for Tenecteplase by Effective Reperfusion of peNumbrAL tissue in patients with Large Vessel Occlusion (ETERNAL LVO)

Multicentre, PROBE, phase 3, parallel group, controlled trial with adaptive covariate adjusted randomisation and adaptive sample size re-estimation.

Protocol No: UOM2102

EudraCT Number: 2021-001635-12

Version: 4.0 Date: 19 October 2021

Sponsor: University of Melbourne

CONFIDENTIAL

This protocol is confidential and is the property of University of Melbourne. No part of it may be transmitted, reproduced, published, or used without prior written authorisation from the institution.

Statement of Compliance

This clinical trial will be conducted in compliance with all stipulation of this protocol, the conditions of the ethics committee approval, the NHMRC National Statement on ethical Conduct in Human Research (2007 and all updates), the Integrated Addendum to ICH E6 (R1): Guideline for Good Clinical Practice E6 (R2), dated 9 November 2016 annotated with TGA comments and the NHMRC guidance Safety monitoring and reporting in clinical trials involving therapeutic goods (EH59, 2016).

This clinical trial will be conducted in compliance with all stipulations of this protocol, the conditions of the ethics committee approval, standards of Good Clinical Practice (as defined by the International Conference on Harmonisation), ethical principles that have their origin in the Declaration of Helsinki and all applicable national and local regulations.>

Protocol Number: UOM2102

Protocol Title: ETERNAL LVO

This clinical trial is not sponsored by any pharmaceutical company or other commercial entity.

AMENDMENTS:

25/07/2019	Section 1 clarification of terms, deletion of text, inclusion/exclusion clarification of occlusions and penumbra volume.<br>Section 4 study design clarification.<br>Section 5 deletion of text and revised penumbra volume.<br>Section 6,7 and 8.4 inclusion/exclusion clarification of occlusions and penumbra volume.<br>Section 15.4 Consent section revised for guardianship.<br>Section 15.7 Notification of primary physician deleted.
22/11/2019	Corrected header version, updated Amendment table with change from version 1 to version 2.<br>Added Appendix 1 Study schedule of events table
26/5/2021	Statement of Compliance clause addition<br>EurdaCT Number for international standards<br>Revised protocol number for international standards.
19/10/2021	Removal of "*Item 6.2 - ' Exclusion criteria:… Unable to gain consent from patient or person responsible"*

Study acknowledgment/confidentiality

By signing this Protocol, the Investigator(s) acknowledges and agrees:

The Protocol contains all necessary details for conducting the study. The Investigator will conduct this study as detailed herein, in compliance with Good Clinical Practice[1] (GCP) and the applicable regulatory requirements, and will make every reasonable effort to complete the study within the time designated.

The Protocol and all relevant information on the drug relating to pre-clinical and prior clinical experience, which was furnished by the Sponsor, Company Name, will be made available to all physicians, nurses and other personnel who participate in the conducting of this study. The Investigator will discuss this material with them to assure that they are fully informed regarding the drug(s) and the conduct of the study.

This document contains information that is privileged or confidential. As such, it may not be disclosed unless specific prior permission is granted in writing by Company Name or such disclosure is required by federal or other laws or regulations. Persons to whom any of this information is to be disclosed must first be informed that the information is confidential. These restrictions on disclosure will apply equally to all future information supplied, which is indicated as privileged or confidential.

Company Name will have access to any source documents from which Case Report Form information may have been generated. The Case Report Forms and other data pertinent to this study are the sole property of, Company Name, which may utilise the data in various ways, such as for submission to government regulatory authorities, or in publication of the results of the study.

The conduct and results of this study will be kept confidential. The results of this study may be published. Upon completion of the Study it is the intention of the parties to prepare a joint publication regarding or describing the Study and all the results there from and both parties shall co-operate in this regard. Where it is the intention of Company Name to file for a patent or other intellectual property right protection, publication may be deferred at the option of Company Name for up to twelve months from the date of completion of the proposed joint publication to allow Company Name to make all filings it deems appropriate.

<u>Investigator Signatory:</u>

Study Chairman:

Mark Parsons		18/03/2019
**Name**	**Signature**	**Date**

Protocol Number: UOM2102

Protocol Title: ETERNAL LVO

Sponsor	Signature	Date

Study Team

CHAIR OF THE STEERING COMMITTEE AND CORRESPONDING AUTHOR
Prof. Mark Parsons
University of Melbourne
Parkville, VIC, Australia 3010

CO-PRINCIPAL INVESTIGATORS AND REGIONAL MEDICAL COORDINATORS

ASIA
Prof. Henry Ma
Monash Medical Centre
Clayton, VIC 3168 AUSTRALIA
henry.ma@monashhealth.org

AUSTRALIA/New Zealand
Prof Bruce Campbell
Royal Melbourne Hospital
Parkville, VIC 3050 AUSTRALIA
bruce.campbell@mh.org.au

NORTH AMERICA AND EUROPE
Prof. Ken Butcher
Prince of Wales Hospital
Randwick NSW 2031 AUSTRALIA

BIOSTATISTICIAN
Prof. Leonid Churilov
Melbourne Medical School
University of Melbourne
Parkville Vic 3050 AUSTRALIA
leonidc@unimelb.edu.au

IMAGING MANAGER
A/Prof Andrew Bivard
Melbourne Brain Centre
University of Melbourne
Parkville Vic 3050 AUSTRALIA
Abivard@unimelb.edu.au

CLINICAL COORDINATOR
Amy McDonald
Melbourne Brain Centre
University of Melbourne
Parkville Vic 3050 AUSTRALIA
amy.mcdonald@mh.org.au

Protocol Authors
Prof Mark Parsons, A/Prof Andrew Bivard, Prof Leonid Churilov, Prof Henry Ma, Prof Ken Butcher, Prof Bruce Campbell, Prof Stephen Davis, and Prof Geoffrey Donnan.

ClinicalTrials.gov Identifier: NCT04454788

Table of Contents

Study acknowledgment/confidentiality 3
Study Team 4
Abbreviations and definitions of terms 7
1. Synopsis 9
2. Background 13
3. Study Objectives 20
4. Study Design 20
5. Study Population 20
6. Number of Subjects 21
6.1 Inclusion Criteria 21
6.2 Exclusion Criteria 21
6.3 Other Eligibility considerations (patients taking oral anticoagulants) 22
6.4 Feasibility 22
7. Study assessments and Procedures 23
7.1 Screening 23
7.2 Study Procedures 24
7.2.1 Day 0 – Treatment 24
7.2.2 Day 1 (24 hours post treatment administration) 24
7.2.3 Day 90 +/- 7 days 24
7.2.4 Day 360 +/- 30 days 25
7.3 Efficacy Assessments 25
7.4 Study Restrictions 25
8. Investigational products 25
8.1 Comparator Justification 25
8.2 Description of Investigational Product 26
8.3 Administration 26
8.4 Randomisation 26
8.5 Blinding/Unblinding 27
8.6 Product Labelling 27
8.7 Handling and Storage of Investigational Product 27
9. Adverse events and Serious adverse events 27
9.1 Definition of an Adverse Event (AE) 27
9.2 Definition of a Serious Adverse Event (SAE) 28
9.3 Clinical Laboratory Abnormalities and Other Abnormal Assessments as AEs and SAEs 29
9.4 Time Period, Frequency, and Method of Detecting AEs and SAEs 30

9.5 Recording of AEs and SAEs 30
9.6 Prompt Reporting of SAEs to the Study Management Centre 30
9.7 Expeditable Events 31
9.8 Evaluating AEs and SAEs 31
9.8.1 Assessment of Intensity 31
9.8.2 Assessment of Causality 32
9.8.3 Assessment of Expectedness 32
9.9 Follow-up of AEs and SAEs 33
9.10 Post-study AEs and SAEs 33
10. Subject completion and discontinuation 33
10.1 Subject Completion 33
10.2 Subject Withdrawal 33
10.3 Discontinuation Criteria 34
11. Case report form 34
12. Data analysis and statistical considerations 34
12.1 Objective 34
12.2 Endpoints 34
12.3 Sample size 35
12.4 Interim/Safety analysis 36
12.5 Statistical analysis 36
13. Data management 37
14. Study monitoring 37
14.1 Curriculum Vitae and Other Documentation 38
14.2 Investigator Responsibility 38
14.3 Study Report 38
15. Administrative Procedures 38
15.1 Ethical Considerations 38
15.2 Ethical Review Committee 38
15.3 Regulatory Authorities 39
15.4 Informed Consent 39
15.5 Subject Reimbursement 39
15.6 Emergency Contact with Investigators ..... **Error! Bookmark not defined.**
15.7 Notification of Primary Care Physician ..... **Error! Bookmark not defined.**
15.8 Investigator Indemnification 40
15.9 Financial Aspects 40
15.10 Protocol Amendments 40
15.11 Protocol Compliance 40
15.12 Archives: Retention of Study Records 41
16 References. 41

17 Appendix 1 Study Schedule of Events Table

Abbreviations and definitions of terms

AE	Adverse Event
ALT (SGPT)	Alanine Transaminase
AST (SGOT)	Aspartate Transaminase
aPPT	Activated Partial Thromboplastin Time
AUC_{0-24}	Area Under the Concentration-Time Curve From Time Zero To 24 Hours
BP	Blood Pressure
BMI	Body Mass Index (Weight In Kg Divided By Height In M^2)
C_{max}	Maximum Plasma Drug Concentration
CARA	Covariate-adjusted response adaptive randomisation
CIB	Clinical Investigators' Brochure
CRF	Case Report Form
CT	Computed Tomography
CTA	Computed Tomography Angiography
CTP	Computed Tomography Perfusion
CTN	Clinical Trial Notification
DSA	Digital Subtraction Angiography
DWI	Diffusion Weighted Imaging
ECG	Electrocardiogram
EQ5D	European Quality of Life assessment with 5 dimensions
FLAIR	Fluid-Attenuated Inversion Recovery
GCP	Good Clinical Practice
GRF	Glomerular Filtration Rate
GGT	Gamma Glutamyl Transpeptidase
GRE	Gradient Echo
GI	Gastrointestinal
ICH	IntraCerebral Haemorrhage
ICH	International Conference on Harmonisation
Hb	Haemoglobin
HCT	Haematocrit
IEC	Independent Ethics Committee
Hr	Hour
LFT	Liver Function Test

LLOQ	Lower Limit of Quantification
MDTS	Metered-Dose Transdermal Spray
MRI	Magnetic Resonance Imaging
NHMRC	National Health and Medical Research Council
NCCT	Non-Contrast Computed Tomography
PROMIS10	Patient-Reported Outcomes Measurement Information System
POC	Point of Care
PWI	Perfusion Weighted Imaging
PK	Pharmacokinetic
SAE	Serious Adverse Event
SD	Standard Deviation
$t_{1/2}$	Terminal Half-Life = Ln 2/Λz
TGA	Therapeutic Goods Administration
TNK	Tenecteplase
t_{max}	Time of Occurrence of C_{max}
ULN	Upper Limit of Normal
US	United States
WBC	White Blood Cells

1. Synopsis

**Study Title**	ETERNAL LVO: Extending the time window for Tenecteplase by Effective Reperfusion of peNumbrAL tissue in patients with Large Vessel Occlusion
**Development phase:**	Phase III
**Indication:**	Acute ischaemic stroke presenting within 24 hours with a large vessel occlusion and mismatch on perfusion imaging.
**Product**	Intravenous tenecteplase (0.25mg/kg) versus 'best practice' which may be alteplase (0.9mg/kg) or standard care (no lysis) at treating clinician's discretion
**No. Subjects:**	The sample size is estimated to be 370 per treatment arm (740 total). Adaptive sample size re-estimation according to Mehta and Pocock promising zone method will occur at 296 per arm (80% of estimated sample size) with a maximum of 500 per arm (1000 total).
**No. Centers:**	Approximately 75 centres worldwide (including telestroke sites). Countries will include Australia, New Zealand, Taiwan, Korea, Canada, United Kingdom, Spain, Finland, Germany, and other EU countries.
**Study Duration:**	Estimated study duration is 5 years. Patients will participate in the trial for 12 months.
**Primary Objective:**	The primary objective of the study is to test the hypothesis that the thrombolytic tenecteplase (TNK, 0.25mg/kg) administered within 24 hours after symptom onset, is superior to current best practice (alteplase, rtPA, 0.9mg/kg or standard care/no lysis) in achieving excellent functional outcome or return to the premorbid modified Rankin Scale at 90 days in acute ischemic stroke patients with a large vessel occlusion and a significant volume of penumbral tissue on multimodal CT or MRI.
**Study design:**	The study will be a prospective, randomised, open-label, blinded endpoint (PROBE), phase 3 parallel group superiority trial, with covariate adjusted randomisation and adaptive sample size re-estimation.<br><br>Study population - ischaemic stroke patients with large vessel occlusion presenting to hospital within 24 hours of symptom onset.<br><br>Patients will be required to have a large vessel occlusion on baseline CT angiography (CTA) or MR angiography (MRA). LVO will be defined as 'potentially retrievable' thrombus at one or more of the following sites: intracranial and/or extracranial internal carotid (ICA), middle cerebral artery (MCA) first segment (M1) and proximal second segment of middle cerebral artery (M2). Endovascular thrombectomy will be allowed but is not mandatory. Additionally, patients must

	have met the CT perfusion defined mismatch criteria (perfusion lesion >15mL, mismatch ratio>1.5 and a baseline ischemic core <70mL)<br><br>Patients will be randomized to treatment with either standard of care (no intravenous thrombolytic treatment or intravenous alteplase 0.9mg/kg) or intravenous tenecteplase (0.25mg/kg), using an adaptive covariate adjusted randomisation procedure to minimise imbalance on the following covariates: clinician intention to treat with alteplase or no thrombolysis treatment if randomised to standard of care, age, NIHSS, premorbid mRS, site of Large Vessel Occlusion site (Intracranial ICA and/or M1 vs extracranial ICA and/or M2, with tandem ICA occlusions to be considered intracranial for the purpose of covariate adjustment), and onset-to-randomization time (with bins for: 0-4.5 hours, 4.5-12 hours and ≥12 hours, where wake-up stroke/uncertain onset is 'last seen well within last 24 hours').
**Study endpoint:**	**Primary Outcome**<br>The proportion of patients with Modified Rankin Scale (mRS) 0-1 (no disability) or return to baseline mRS (if baseline premorbid mRS =2) at 3 months.<br><br>**Secondary Outcomes**<br>*Efficacy*<br>• Proportion of patients achieving early clinical improvement (reduction in acute – 24 hour NIHSS score of ≥8 or 24 hour NIHSS 0-1).<br>• Proportion of patients with Modified Rankin Scale 0-2 at 3 months<br>• Ordinal analysis of mRS at 3 months<br>• Proportion of patients with TICI 2b/3 on initial digital subtraction angiography run prior to thrombectomy<br>*Safety*<br>• Proportion of patients with symptomatic intra-cerebral haemorrhage (sICH) defined as: parenchymal haematoma type 2 (PH2) within 36 hours of treatment combined with neurological deterioration leading to an increase of ≥4 points on the NIHSS from baseline.<br>• Proportion of patients with death due to any cause<br>• Proportion of patients with Modified Rankin Scale (mRS) 5-6 at 3 months (severe disability or death).<br>*Exploratory outcomes*<br>• Proportion of patients achieving reperfusion (>90% and >50%) at 24 hours post stroke<br>• Infarct growth at 24 hours<br>• Proportion of patients achieving recanalisation at 24 hours post stroke (CTA or MRA).
**Inclusion criteria:**	• Patients presenting with acute hemispheric ischaemic stroke with onset (or the time last known to be well) within 24 hours.<br>• Patient's age is ≥18 years

	• Premorbid mRS <3, with a concurrent assessment of whether the patient was able, immediately prior to the stroke, to: 1) Drive, or (if never drives) perform own Domestic duties, and 2) Shop for themselves, and 3) Bank/do their own finances (i.e. Drive/Domestic, Bank, Shop = DBS +ve). Need to be DBS +ve to be study eligible.<br>• Presence of a large vessel occlusion on CTA or MRA. LVO will be defined as 'potentially retrievable' thrombus at one or more of the following sites: extracranial or intracranial internal carotid (ICA), middle cerebral artery (MCA) first segment (M1) or proximal middle cerebral artery second segment (M2). Presence of 'target mismatch' on perfusion CT (CTP) or diffusion-perfusion MRI as processed by MIStar. Mismatch is defined as an ischaemic core of <70mL, penumbra of >15mL, and an ischemic core to perfusion lesion ratio of >1.8.
**Exclusion criteria:**	• Intracranial hemorrhage (ICH) or other diagnosis (e.g. tumor) identified by baseline imaging.<br>• Basilar artery occlusion, extensive early ischaemic change (hypodensity on NCCT) and/or early ischaemic change outside the perfusion lesion that invalidates mismatch criteria.<br>• Pre-stroke mRS of > 2 (indicating significant previous disability) or DBS –ve (i.e. was unable to perform one of the DBS items).<br>• Any terminal illness such that patient would not be expected to survive more than 1 year<br>• Any condition that, in the judgment of the investigator could impose hazards to the patient if study therapy is initiated or affect the participation of the patient in the study.<br>• Clinically evident pregnant women.<br>• Other standard contraindications to thrombolysis.<br>• Minor stroke symptoms, or major stroke symptoms rapidly improving<br>• Clinical presentation suggesting subarachnoid haemorrhage<br>• Known bleeding diathesis and/or platelet count <100 000, or taking warfarin with INR > 1.7.<br>• Patients who have received heparin within 48 hours must have normal aPTT.<br>• Major surgery or serious trauma within 14 days, serious head trauma within 3 months.<br>• GI or urinary tract haemorrhage within last 21 days<br>• Arterial puncture at a non-compressible site or lumbar puncture within 7 days<br>• Systolic BP > 185, diastolic BP > 110mmHg<br>• Clinical stroke within 3 months or history of ICH<br>• Known severe renal impairment (GFR < 15mls/min)
**Study procedures:**	1. Baseline multimodal CT (NCCT, CTA, and CTP) or MRI (DWI, PWI, GRE/SWI, FLAIR), NIHSS and clinical assessments to assess for thrombolysis and study eligibility.

	2. Randomisation and treatment as soon as possible after baseline imaging.<br>3. Study MRI (DWI, FLAIR, GRE or SWI, and PWI), or multimodal CT (NCCT, CTA, CTP) at 24 hours after randomization to measure infarct volume, reperfusion, recanalisation.<br>4. NIHSS at 24 hours post randomisation and clinical observations<br>5. Modified Rankin Scale (mRS) and Quality of Life assessment (EQ5D and PROMS10) – at 3 months<br>6. Modified Rankin Scale (mRS) and Quality of Life assessment (EQ5D and PROMS10) – at 12 months
**Study sample size and statistical considerations:**	An estimated total sample size of 740 patients (equally distributed between two study arms) will yield 80% power to detect an absolute difference of at least 10% in proportion of patients achieving the primary outcome between two arms using two-sided statistical significance threshold of p=0.05. The estimates in the 'current best practice' group are based on the outcomes of the EXTEND trial where the proportions of LVO patients with the primary outcome (3-month mRS 0-1) were: placebo arm 25%, alteplase arm 28%, with the more conservative assumed proportion taken as 28%. The treatment effect of tenecteplase was based upon the pooled analysis of phase II tenecteplase LVO data where the tenecteplase treatment effect size was 19% greater than alteplase. A more conservative treatment effect of 10% has been assumed for ETERNAL.<br><br>Adaptive increase in sample size is planned if the result of interim analysis using data from the first 592 patients are promising, as per the methodology of Mehta and Pocock. The maximum sample size is capped at 1000 patients (500 per arm).<br><br>The primary analysis will be conducted on intention to treat basis. A detailed statistical analysis plan will be formulated before the database lock. The analysis of the primary outcome will be conducted using a modified Poisson regression with excellent functional outcome (mRS 0-1) or return to the pre-morbid mRS as the dependent variable, treatment group as the independent variable and age, NIHSS and onset-to-ECR puncture time (categorized as no ECR, <2 hours, and > 2 hours) as treatment covariates. Secondary and exploratory analyses will be undertaken using appropriate regression models.

2. Background

Ischaemic stroke is the leading cause of adult disability in the world.[1] The current standard of care for treatment of patients with acute ischaemic stroke is intravenous recombinant tissue plasminogen activator (tPA, or alteplase), although its use currently remains restricted to less than 4.5 hours from symptom onset. Presently, only 13%[2] of ischaemic stroke patients in Australia receive alteplase, partly due to the time window restriction. Additionally, alteplase performs relatively poorly in patients with a large vessel occlusion (LVO).[3] Despite availability of alteplase, stroke patients with LVO face a devastating outcome due to the large area of brain tissue affected, resulting in >50% of patients dead or highly disabled at 3 months.[4] However, recent trials have shown that endovascular clot retrieval or thrombectomy (EVT) results in at least a doubling of the odds of disability-free survival, compared to treatment with alteplase alone.[5,6,7] It is therefore no surprise that there has been worldwide enthusiasm to translate EVT into routine practice. However, endovascular thrombectomy requires considerable operator expertise and infrastructure, so is limited to high volume stroke centres (which also ensures lower complication rates). This means there are inevitable delays in LVO patients receiving EVT, particularly in rural and regional centres, but also in large cities where inter-hospital transfers can be slow. Intravenous thrombolysis given prior to transfer to a thrombectomy capable centre ('drip and ship') may lead to vessel recanalisation in transit and negate the need for thrombectomy. However, alteplase only leads to recanalization prior to thrombectomy in 10% of patients.[8] Further, its use is currently restricted to <4.5 hours after symptom onset.

"Tissue is more important than time"

Although it remains essential to treat acute ischaemic stroke patients with reperfusion therapy (intravenous thrombolysis and/or EVT) as quickly as possible, years of research by our group, and others, have now clearly demonstrated that the treatment window can be safely extended using modern brain imaging.[9,10] Initially, MRI was used to define salvageable brain tissue (penumbra) and dead brain tissue (ischemic core), but now multimodal CT (incorporating non-contrast CT, CT angiography, and perfusion CT) allows similar assessment of tissue pathophysiology, and is much more accessible around the world, including in rural and regional Australia. Perfusion CT (CTP) is capable of characterizing tissue viability by measuring hemodynamic changes in ischaemic tissue. The ischaemic penumbra can be quantitatively localized on CTP by setting specific physiological cut-offs for the various perfusion maps (also referred to as 'thresholds'). Using these thresholds, it is possible to estimate the volume and location of irreversibility

damaged tissue (the ischemic core) and potentially salvageable ischemic tissue (penumbra). As a result of its ability to positively identify ischemia as well as core and penumbra, CTP has been implemented into routine practice in many centres around the world.

Our group has been integral to the implementation of CTP into routine clinical practice, which has accelerated with specific software applications now being available that automate the processing of perfusion data using validated algorithms and distribute the results via email and picture archiving and communication systems (PACS). Individual reperfusion treatment response is highly variable due to many factors, but the most important predictor of treatment response is how much dead brain (ischemic core) is present, and how much ischemic brain tissue (penumbra) is left to salvage at the time of delivering treatment. If brain tissue is already irreversibly injured, restoration of blood supply will not improve clinical outcome of the individual patient, and reperfusion therapy is futile.[11] There are also potential risks of reperfusion injury when extensive irreversible injury is already established – haemorrhagic transformation and malignant oedema. Multi modal imaging with computed tomography perfusion (CTP) has now been well validated to identify subgroups of patients with varying treatment responses to reperfusion therapy.

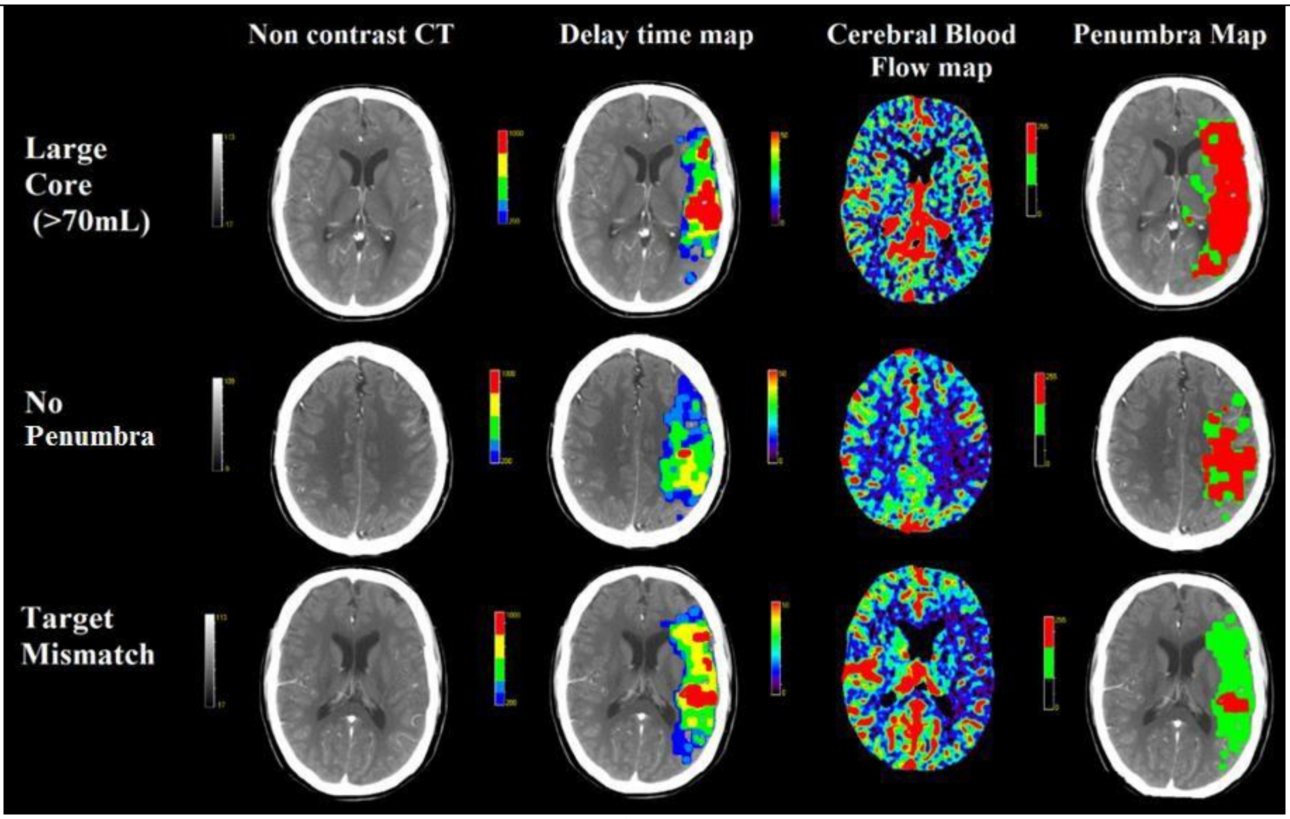

Figure 1.Using multimodal CT, we have identified three groups whose response to intravenous thrombolysis is quite different. Patients with large established ischemic core do not benefit and may be harmed thrombolysis due to excessive bleeding risk (First row, large ischemic core >70mL). Patients who do not have a large ischemic core but have no significant tissue left to salvage (second row, no mismatch). Finally, patients with a small ischemic core and large penumbra who benefit substantially (third row, target mismatch). These patient groups cannot be differentiated by standard clinical criteria or by non-contrast CT imaging (first column).

Our group has also led the introduction and implementation of automated processing of CTP data (figure 1) to provide real-time ischemic core and penumbral volumes. This technology has already been implemented in many stroke centres around Australia and the world (with our colleagues in the United Kingdom, Spain, Taiwan, Finland, and Canada) and is being used in both routine practice and for clinical trials (TASTE trial).

Following on from validation work by our group, the tissue selection approach using multimodal CT has recently been used to extend the treatment window for endovascular thrombectomy to 24 hours.[9,10] Additionally, we have recently completed the first trial ever to show that the time window can be extended for intravenous thrombolysis. EXTEND[12] was a randomised double-blind placebo-controlled trial and demonstrated a clinical benefit in patients treated with intravenous alteplase beyond 4.5 hours and up to 9 hours after stroke onset (including 'wake-up stroke'). A crucial element of the EXTEND trial design was imaging selection of patients with a small core and significant volume of penumbra using

automated image processing to standardize interpretation. Together with the DAWN and DEFUSE3 thrombectomy trials, these clinical trials demonstrated that patients do receive benefit from reperfusion treatment in an extended time window *if the patient has a small ischemic core and a significant volume of salvageable brain tissue*.

Although EXTEND was a positive study, the unadjusted absolute benefit of treatment from alteplase in terms of excellent functional outcome at 3 months in the subgroup of patients with large vessel (LVO) patients was very small (25% placebo versus 28%). Similarly, both DAWN and DEFUSE3 had low rates of good 3 month outcome (<20%) in the standard care group (where only 10% of patients received IV thrombolysis due to the late time window). Clearly, outcomes in LVO patients with onset beyond 4.5 hours could be considerably improved upon by a more effective thrombolytic. In terms of patient numbers and potential impact on stroke outcomes, *more than 40% of all referrals for thrombectomy in 2018 to Royal Melbourne Hospital were for patients beyond 6 hours and 66% of patients were transferred to the Royal Melbourne Hospital from regional areas*. Based on current guidelines, none of these patients received IV thrombolysis prior to transfer.

Our group has led the world in testing what appears to be a more effective thrombolytic agent[13,14] for ischaemic stroke. Tenecteplase (TNK) is a genetically engineered mutant tissue plasminogen activator that has a longer half-life (~22 minutes vs ~3.5min with alteplase, allowing single bolus administration rather than a 1 hour infusion with alteplase), is more fibrin-specific, is more resistant to plasminogen activator inhibitor-1 than alteplase, and does not exhibit the in vitro neurotoxicity that has been observed with alteplase. These pharmacologic and practical advantages make tenecteplase an attractive alternative to alteplase, in particular for inter-hospital transfers. Several trials have already been completed with tenecteplase in acute stroke to provide firm grounding for the treatment dose and establish a safety profile.[15,16]

After an initial pilot study[17] our group compared 0.9mg/kg alteplase to two doses of TNK (0.10mg/kg and 0.25mg/kg) in a phase II randomised trial that required patients to have a ‘dual target’ (vessel occlusion on CT angiography and CTP evidence of small core and salvageable brain). The trial demonstrated that treatment with 0.25mg/kg resulted in significantly increased reperfusion at 24 hours and better early neurological recovery, which also translated into improved 3 month outcomes.[13] Of note, this study contained only LVO patients, yet the 24 hour reperfusion rates with TNK were similar to those seen with endovascular thrombectomy in our EXTEND IA study, which was one of the landmark studies leading to EVT becoming the standard of care for LVO stroke.[18]

Figure below

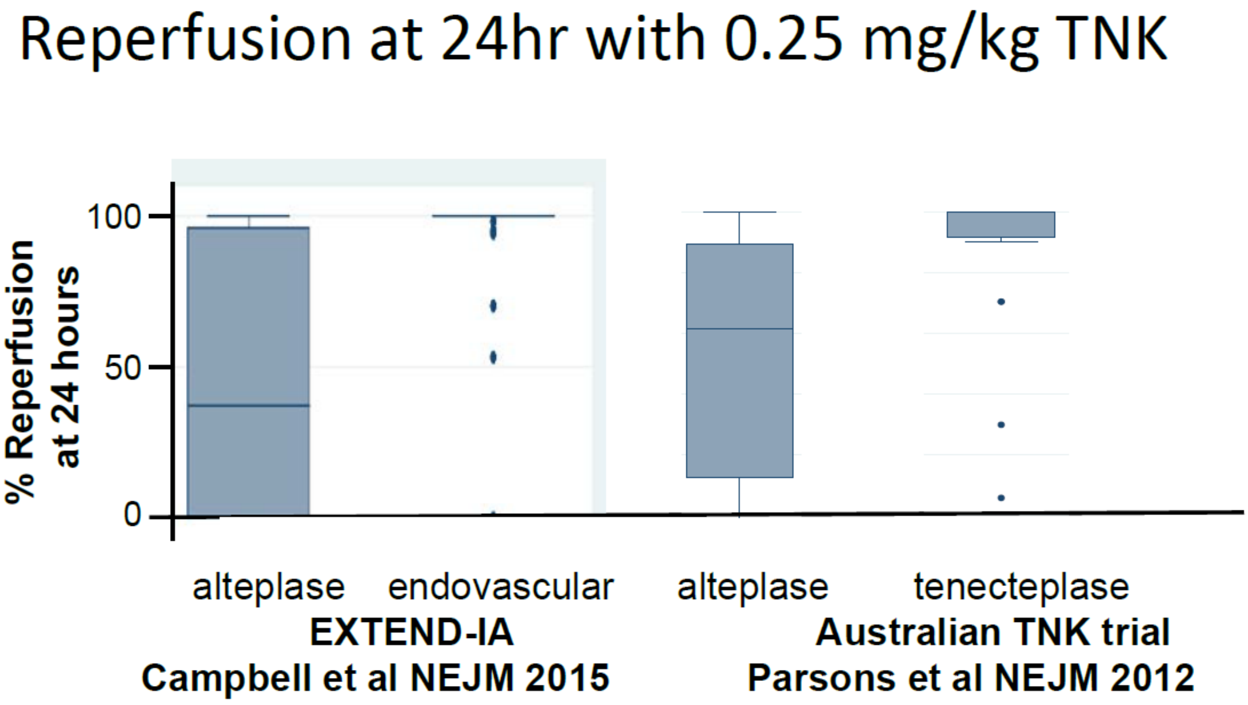

The benefit of identifying ischemic tissue and salvageable tissue prior to reperfusion therapy and by excluding mimics using CTP[19] was highlighted by a subsequent phase II tenecteplase trial (ATTEST), which enrolled patients via standard clinical criteria. ATTEST was negative for the primary outcome. Although ATTEST did not use CTP to select patients, fortunately, CTP was still performed. When the ATTEST data was pooled with our Australian TNK trial, only 34% of ATTEST patients had salvageable tissue meeting 'target mismatch' criteria on CTP (explaining the differing results). In the pooled data, there was a substantial clinical benefit over alteplase in patients treated with tenecteplase who had salvageable tissue (return to all usual activities [mRS 0-1] occurred in 35% tenecteplase vs 22% alteplase-treated patients), *with a lower brain haemorrhage rate*.[20]

The recent EXTEND-IA TNK trial, also by our group, showed that 0.25mg/kg tenecteplase was more effective than the standard stroke thrombolytic drug alteplase in patients treated up to 4.5 hours from symptom onset at opening large vessel occlusions prior to thrombectomy in patients who met target mismatch criteria on CTP. The primary outcome of reperfusion at the initial angiogram occurred in 22% of tenecteplase versus 10% of alteplase patients (p=0.02). Thus, *1 in 5 LVO patients treated with tenecteplase did not require subsequent thrombectomy* and the number needed to treat with tenecteplase rather than alteplase to prevent one thrombectomy was nine. Although all patients with persisting LVO still went on to thrombectomy, tenecteplase was also associated with improved functional outcome at 90 days (common odds ratio 1.7, 95%CI 1.0-2.8, p=0.037, Figure 3). This emphasises the benefits of earlier reperfusion with tenecteplase.

Thrombectomy access in regional areas. 'Drip and ship' in an extended time window.

Thrombectomy is a highly resource intensive (people and infrastructure) therapy and therefore thrombectomy capable centres are limited to a few metropolitan hospitals in Australia. Direct access to thrombectomy is even more limited worldwide. Given the results of DAWN and DEFUSE3, it is now possible to treat CTP or MR-selected patients up to 24 hours after symptom onset, and thus thrombectomy centres are accepting many more transfer cases than when the treatment window was <6 hours. This is particularly relevant since people living in regional Australia are 19 percent more likely to have a stroke.[21] As part of the transfer protocol, LVO patients within 4.5 hours are given IV alteplase in the regional centre and 'shipped' to a thrombectomy centre. Unfortunately, alteplase performs poorly in this population, as shown in many studies including our recent EXTEND IA TNK trial. Additionally, patients outside the proven 4.5 hours thromolysis time window are being 'shipped' for thrombectomy without any 'drip'. This means sometimes many hours lapse before recanalization can be achieved via delayed thrombectomy with conversion of penumbra on initial imaging into ischemic core by the time thrombectomy occurs. Notably, our recent EXTEND trial (in which subsequent thrombectomy was not performed) showed only a marginal benefit of alteplase compared to placebo in LVO patients. *The data for tenecteplase compared to alteplase in LVO patients is compelling (early reperfusion prior to angiogram of 10% with alteplase vs 22% with tenecteplase),* with a higher rate of early reperfusion and corresponding better patient outcomes. However, these data are all from the early time window (<4.5 hours). There is no reason to suspect tenecteplase would not lead to similar improved outcomes if given to patients with target mismatch beyond 4.5 hours. Indeed, the effect may be amplified with longer delays seen between administration of tenecteplase and thrombectomy in regional patients. The reperfusion rates in EXTEND IA TNK reflected treatment at endovascular-capable centres in the majority of patients, with only 25% undergoing inter-hospital transfer within the metropolitan area (median thrombolysis to arterial puncture time 43min). As such, TNK may not have had 'long enough to work'. Our phase II TNK data supports this by showing similar 24 hour reperfusion rates with TNK to endovascular therapy (and considerably superior to alteplase).

What about tenecteplase in the 0-4.5 hour window?

Although both of our previous early time window studies showed a significant benefit of tenecteplase compared to alteplase in terms of 3-month outcome, they were relatively small phase II studies. Our team is running a large phase III trial of tenecteplase versus alteplase (TASTE) which has enrolled over 360 patients (target 700), but it excludes patients with LVO in whom thrombectomy is planned. There is no phase III trial enrolling LVO patients to tenecteplase versus alteplase. Thus, we plan to include LVO patients in the 0-4.5 hour window in ETERNAL. Given the data we have so far, where tenecteplase leads to earlier reperfusion, combined with the low rates of reperfusion with alteplase in LVO, tenecteplase may be particularly beneficial in 'drip and ship' patients where there is a delay between thrombolysis and thrombectomy.

Thus, it is now time to definitively assess tenecteplase in LVO patients in both the early (0-4.5 hours) and delayed (4.5 – 24 hour) time window. ETERNAL LVO will be a phase III trial of treatment with 0.25mg/kg tenecteplase compared to 'standard of care' in patients with a large vessel occlusion and target mismatch on CTP between up to 24 hours after symptom onset. Following publication of our EXTEND trial results we expect there will be some variation in the standard of care for patients with target mismatch in the late time window and anticipate that some clinicians will now wish to give alteplase. However, given that the EXTEND results, as a single trial, may not be sufficient to generate a strong guideline recommendation regarding administration of thrombolysis beyond 4.5 hours, we expect variation in clinical practice in terms of whether 'standard of care' for LVO patients beyond 4.5 hours includes IV alteplase or no thrombolytic. To accommodate this expected variation in practice, the 'standard of care arm' will allow either 0.9mg/kg alteplase or no lytic at the local investigator's discretion, but with the control group treatment pre-specified by the investigator (i.e. alteplase or not) prior to randomisation (covariate adjusted randomisation to minimise imbalances in key prognostic covariates between treatment groups). For example, investigators may be more likely to choose no lytic in patients with larger ischaemic cores (due to perceived risk of ICH), or in those treated in the later part of the 24 hour time window. If not adjusted for at randomisation, this might lead to an imbalance for these variables between the tenecteplase and 'standard of care' groups.

3. Study Objectives

The primary objective of the study is to test the hypothesis that the thrombolytic tenecteplase (TNK, 0.25mg/kg) administered within 24 hours after symptom onset, is superior to current best practice (alteplase, rtPA, 0.9mg/kg or standard care/no lysis) in achieving excellent functional outcome or return to the premorbid modified Rankin Scale at 90 days in acute ischemic stroke patients with a large vessel occlusion and a significant volume of penumbral tissue on multimodal CT or MRI.

The primary hypothesis for ETERNAL LVO is that patients treated with 0.25mg/kg tenecteplase will have a greater proportion of Modified Rankin Scale (mRS) 0-1 (no disability) or return to baseline mRS (if baseline premorbid mRS =2) at 3 months compared to those treated with standard of care (0.9mg/kg alteplase or placebo).

4. Study Design

The study will be a prospective, randomised, open- label, blinded endpoint (PROBE), phase 3, parallel group, superiority trial with covariate-adjusted 1:1 randomisation, and adaptive sample size re-estimation in ischemic stroke patients with large vessel occlusion presenting to hospital within 24 hours of symptom onset (or last known well within 24 hours). Patients will be randomised to treatment with either standard of care (no intravenous thrombolytic treatment or intravenous alteplase 0.9mg/kg at the investigators discretion) or intravenous tenecteplase (0.25mg/kg). A covariate-adjusted randomisation procedure will be used to minimise imbalances for the following covariates: clinician intention to treat with alteplase or no thrombolysis if randomised to standard of care arm, age, NIHSS, premorbid mRS, site of Large Vessel Occlusion (Intracranial ICA and M1 vs extracranial ICA and M2, with tandem ICA occlusions to be considered intracranial for the purpose of covariate adjustment), and onset-to-randomisation time (less than 4.5h, 4.5-12h and 12-24h, where wake-up stroke/uncertain onset is 'last seen well within last 24 hours').

5. Study Population

The ETERNAL LVO trial will include patients with acute hemispheric ischaemic stroke, who are ≥18 years of age and are eligible for standard intravenous therapy within 24 hours of stroke onset or last known well within 24 hours, who have a large vessel occlusion. In addition to standard thrombolysis eligibility criteria, patients will be assessed for the presence of salvageable tissue without a large ischaemic core on CTP or MRI. Those that are shown by CTP/MRI to have a penumbral volume of greater than 15 mLs, an ischemic core volume of less than or equal to 70 mL, and a 'mismatch ratio' where the ratio of the perfusion lesion volume compared to the ischemic core volume is > 1.8 measured using MIStar software. Large vessel occlusion on pre-randomisation CTA/MRA is *also* a requirement for inclusion into the study.

6. Number of Subjects

The sample size for the ETERNAL LVO study is estimated to be 370 per treatment arm (740 total). Adaptive sample size re-estimation using the Mehta and Pocock promising zone method will be used at n=592 (80% of estimated sample size).

Adaptive increase in sample size will be performed if the result of the interim analysis using data from the first 592 patients is promising. The maximum sample size is capped at 1000 patients.

This sample size is to be achieved through recruitment in Australia, New Zealand, Canada, Taiwan, Korea Germany, Spain, Belgium, Sweden and the United Kingdom at between 50 and 75 selected centres. Many of centres that are primary stroke centres with thrombectomy capability (i.e. 'drip and ship sites). Some of the primary stroke centres will include telestroke sites across Australia and Germany. Other countries may be added.

6.1 Inclusion Criteria

- Patients presenting with acute hemispheric ischaemic stroke with onset (or the time they last known to be well) within 24 hours.
- Patient's age is ≥18 years.
- Premorbid mRS <3, with a concurrent assessment of whether the patient was able, immediately prior to the stroke, to: 1) Drive, or (if never drives) perform own Domestic duties, and 2) Shop for themselves, and 3) Bank/do their own finances (i.e. Drive/Domestic, Bank, Shop = DBS +ve). Need to be DBS +ve to be study eligible.
- Presence of a vessel occlusion on CTA or MRA. LVO will be defined as 'potentially retrievable' thrombus at one or more of the following sites: extracranial and/or intracranial internal carotid (ICA), middle cerebral artery (MCA) first segment (M1) and/or proximal middle cerebral artery second segment (M2).
- Presence of 'target mismatch' on perfusion CT (CTP) or diffusion-perfusion MRI as processed by MIStar. Mismatch is defined as an ischaemic core of <70mL, penumbra of >15mL and an ischemic core to perfusion lesion ratio of >1.8.

6.2 Exclusion Criteria

- Intracranial hemorrhage (ICH) or other diagnosis (e.g. tumor).
- Basilar Artery occlusion.
- Extensive early ischaemic change (hypodensity on NCCT or high signal on DWI-MRI) or early ischaemic change outside the perfusion lesion that invalidates mismatch criteria.
- Pre-stroke mRS score of > 2 (indicating significant previous disability) or DBS –ve.
- Any terminal illness such that patient would not be expected to survive more than 1 year

- Any condition that, in the judgment of the investigator could impose hazards to the patient if study therapy is initiated or affect the participation of the patient in the study.
- Pregnant women.
- Other standard contraindications to thrombolysis.
- Minor stroke symptoms, or major stroke symptoms rapidly improving
- Clinical presentation suggesting subarachnoid haemorrhage
- Known bleeding diasthesis and/or platelet count <100,000 or taking warfarin with INR > 1.7.
- Patients who have received heparin within 48hours must have normal aPTT.
- Major surgery or serious trauma within 14 days, serious head trauma within 3 months.
- GI or urinary tract haemorrhage within last 21 days
- Arterial puncture at a non-compressible site or lumbar puncture within 7 days
- Systolic BP > 185, diastolic BP > 110mmHg
- Clinical stroke within 3 months or history of ICH
- Known severe renal impairment (GFR < 15mls/min)

6.3 Other Eligibility considerations (patients taking oral anticoagulants)

- Warfarin - thrombolysis can be used if POC INR≤1.4.or laboratory INR≤1.7
- Dabigatran – order urgent aPTT, and thrombin time. If dabigatran is known or suspected to have been taken within last 48 hours then Idarucizumab 5g IV bolus should be given prior to thrombolysis.
- Apixaban/Rivaroxaban - If Apixaban/Rivaroxaban is known to have been taken in last 12 hours then patient cannot be enrolled. If unclear, order urgent aPTT, INR, anti-Xa level: patient can be enrolled if appropriately calibrated anti-Xa level indicates <10 ng/mL apixaban or <100 ng/mL rivaroxaban.

6.4 Feasibility

We are uniquely well-placed in the international stroke trials space to rapidly address this urgent knowledge gap and implement substantive changes in clinical practice. We have built an established network of collaborating hospitals across Australia, New Zealand, Taiwan, Korea, Canada, Spain, Finland, Sweden and the UK to recruit acute stroke patients into acute therapy trials. These sites have participated in a number of our trials including TASTE, EXTEND, and EXTEND-IA. Further, we now have a large network of rural/regional telestroke sites in Australia that are actively enrolling in trials such as TASTE and EXTEND-IA TNK. This is important as we expect that >50% of patients recruited in ETERNAL will be 'drip and ship', and, that the group with delays to thrombectomy may stand to gain more benefit from a more effective IV thrombolytic given prior to transfer. Recruitment is expected to take 5 years.

7. Study assessments and Procedures

7.1 Screening

Baseline physical examination will be performed on potentially eligible patients.

Multi-modal CT or MRI will be performed to assess eligibility for acute thrombolytic and thrombectomy treatment including perfusion imaging analysed with MiStar as part of standard care for stroke patients. It is expected the majority of patients will be recruited using CT.

Neurological impairment and functional scores (NIHSS, pre-stroke mRS) will be assessed by a neurologist or trained health care professional.

Bloods for standard care diagnostic evaluations will be collected and analysed.

Patients will be assessed for trial eligibility according to the Inclusion/Exclusion criteria including processing of the imaging data to assess for vessel occlusion and mismatch.

Informed consent will be obtained from patients or their authorised representative according to the Independent Ethics Committee (IEC) approved patient information and informed consent procedure and documentation which may include consent to continue participation after initial "emergency treatment to prevent serious damage to the patient's health" in some jurisdictions.

Imaging: Centres will process the CT or MR images using MIStar to determine eligibility. Patients will have standardised multimodal CT or MR prior to treatment.

Medical history, concomitant medications & ECG: Details of patients' medical history (including stroke history) and concomitant medications will be recorded. Results of ECG performed as part of standard care will be recorded if available.

Blood and urine collection: Results from standard care blood tests performed at screening will be recorded (routine haematology, biochemistry and coagulation screening tests as described in Appendix 1. Study schedule).

Randomisation: Patients eligible for this RCT based on imaging criteria will be randomised to ETERNAL LVO in ratio 1:1 to receive either standard of care or intravenous tenecteplase using a with covariate-adjusted randomisation procedure to minimise imbalances on the following covariates: clinician intention to give alteplase or no thrombolysis if randomised to standard of care, age, NIHSS, premorbid mRS, site of Large Vessel Occlusion (Intracranial ICA and M1 vs extracranial ICA and M2, with tandem ICA occlusions to be considered intracranial for the purpose of covariate adjustment), and onset-to-randomization time (0-4.5h, 4.5-12h, 12-24h, where wake-up stroke/uncertain onset is 'last seen well within last 24 hours').

7.2 Study Procedures

7.2.1 Day 0 – Treatment

Intravenous thrombolytic treatment: Patients will receive open-label intravenous tenecteplase at a dose of 0.25 mg/kg, given as a bolus or 'standard of care', which will include intravenous alteplase at the standard dose of 0.9 mg/kg up to a maximum of 90mg (10% as bolus and the remainder over 1 hour), or no thrombolytic. Randomisation will be performed as described above. The time of bolus and infusion commencement will be recorded.

Close neurological observation will be conducted primarily during the first 48 hours after treatment administration according to local clinical practice.

7.2.2 Day 1 (24 +/- 6 hours post treatment administration)

Imaging: All patients will have MRI (Including DWI, FLAIR SWI/GRE, PWI, and MRA) or, if unable to have MRI, multimodal CT (NCCT with CTA and CTP) at 24 hours post treatment to assess for reperfusion, ICH, infarct growth, and recanalisation.

For all patients who undergo thrombectomy, all DSA imaging will also be required to be uploaded to the ETERNAL eCRF.

Any additional patient imaging acquired during their hospital stay will also be required to be uploaded. Such imaging may include additional NCCT if there is an adverse event or change in neurological status.

Clinical assessments: Neurological assessment (NIHSS) will be performed at 24hrs by an accredited observer who is blind to acute treatment and imaging findings.

Adverse Events: All patients will be asked to report adverse events (refer to Section 8).

Concomitant medications: All concomitant medications will be recorded.

7.2.3 Day 90 +/- 7 days

Clinical assessments: As this is a PROBE (PRospective Open-label Blinded Endpoint) design trial, the primary outcome will be measured by a phone-based assessment of mRS, EQ5D and PROMIS10 from a central agency in each country. Structured questionnaires will be followed. The phone-based assessors will be blind to treatment allocation.

Adverse events: Adverse event data will not be collected unless for mortality, or events serious in nature. Information on stroke recurrence will be collected.

7.2.4 Day 360 +/- 30 days

Clinical assessments: As this is a PROBE (PRospective Open-label Blinded Endpoint) design trial, the primary outcome will be measured by a phone-based assessment of mRS, EQ5D and PROMIS10 from a central agency in each country. Structured questionnaires will be followed. The phone-based assessors will be blind to treatment allocation.

7.3 Efficacy Assessments

All enrolled patients will undergo follow-up MRI or CT at 24 hours after treatment. Clinical assessments will be at baseline and 24 hours (NIHSS), as well as day 90 modified Rankin Scale (mRS, EQ5D and PROMIS10). The 24 hour NIHSS will be measured by an observer blind to treatment allocation and not involved in the patient's baseline assessment or clinical care. Day 90 and day 360 mRS, EQ5D and PROMIS10 will be performed centrally by a trained assessor via telephone (who is blinded to treatment).

7.4 Study Restrictions

- Dietary - Not Applicable
- Smoking – Not Applicable
- Confinement - Not eligible
- Position / Ambulation – patients to be on strict bed rest for 24 hours following administration of trial medications.
- Concomitant Medication – patients not to receive any anti-coagulant/ anti-platelet medications in the first 24 hours following administration of trial medication
- Other Restrictions - Nil

8. Investigational products

For more detail on tenecteplase See Appendix 1.

8.1 Comparator Justification

The ETERNAL LVO study seeks to investigate the clinical benefit of tenecteplase therapy in addition to thrombectomy compared to standard of care. Alteplase is the standard of care treatment for acute ischaemic stroke patients presenting within 4.5 hours of symptom onset. However, in the extended time window, there is not strong evidence for use of IV thrombolysis. In LVO patients, our recent EXTEND study showed only a 3% absolute increase in mRS 0-1 at 3 months with alteplase compared to placebo (unadjusted). Following our EXTEND results we expect there will be some variation in the standard of care for such patients with target mismatch in the late time window and anticipate that some clinicians will now wish to give alteplase. However, given that the EXTEND results, as a single trial, may not be sufficient to generate a strong guideline recommendation regarding administration of thrombolysis beyond 4.5 hours, we expect variation in clinical

practice in terms of whether ‘standard of care’ for LVO patients beyond 4.5 hours includes IV alteplase or no thrombolytic. To accommodate this expected variation in practice the ‘standard of care arm’ will allow either 0.9mg/kg alteplase or no lytic at the local investigator’s discretion, but with the control group treatment pre-specified by the investigator (i.e. alteplase or not) prior to randomisation (covariate adjusted randomisation to minimise imbalances in key prognostic covariates between treatment groups. For example, investigators may be more likely to choose no lytic in patients with larger ischemic core volumes (due to perceived risk of ICH), or in those treated later in the 24 hour time window. If not adjusted for at randomisation, this might lead to an imbalance for these variables between the tenecteplase and ‘standard of care’ groups.

8.2 Description of Investigational Product

The investigational product tenecteplase (Metalyse™, Boehringer Ingelheim) is a genetically modified form of alteplase. Within this study, tenecteplase lyophilised powder will be reconstituted in a glass vial with water for injection at concentration 5mg/mL (e.g. 40mg tenecteplase in 8mL water). Vials should be maintained at a temperature less than 30°C and protected from light as per the manufacturer’s product information. Investigational use of Tenecteplase in this study will be managed under the applicable Regulatory mechanism for each participating country. Off the shelf tenecteplase will be used in this study. Staff will be trained in the mixing and administration of the drug.

8.3 Administration

After reconstitution of the investigational product, a dedicated IV cannula should be used for administration. The dose of tenecteplase to be administered is given as a bolus over approximately 5 seconds. The investigational product should be used immediately after reconstitution. No other anticoagulants or antiplatelet agents are to be given within 24 hours of administration of the investigational product.

8.4 Randomisation

Patients will be randomized according to a centralised web-based procedure coordinated via the Melbourne Brain Centre.

The randomization system will be based on computer generated randomization code lists, using a covariate-adjusted randomisation procedure to minimise imbalances on the following covariates: clinician intention to give alteplase or no thrombolysis if randomised to standard of care, age, NIHSS, premorbid mRS, site of Large Vessel Occlusion (Intracranial ICA and M1 vs extracranial ICA and M2, with tandem ICA occlusions to be considered intracranial for the purpose of covariate adjustment) and onset-to-randomization time (0-4.5, 4.5-12 and 12-24 hours, where wake-up stroke/uncertain onset is ‘last seen well within last 24 hours’).

8.5 Blinding/Unblinding

The investigational treatment is open-label. All those involved in the subsequent clinical and imaging assessment of outcomes will be blinded to treatment allocation. The Data Safety Monitoring Board (DSMB) will have access to unblinded grouped data.

8.6 Product Labelling

Commercial packages of tenecteplase (Metalyse^{TM}, Boehringer Ingelheim) are labelled by the manufacturer, Boehringer Ingelheim, with storage conditions (store below 30°C), batch number and expiry date. Commercial packages of tenecteplase (Metalyse^{TM}, Boehringer Ingelheim) will be used for the Investigational product for this study and be provided by each participating Site under the applicable Regulatory mechanism. Commercial packages are labelled by the manufacturer, Boehringer Ingelheim, with storage conditions (store below 30°C), batch number and expiry date.

8.7 Handling and Storage of Investigational Product

Investigational product will be stored below 30°C until use in accordance with manufacturer's instructions. The investigator or his/her designee must maintain an adequate record regarding the administration of all investigational product within the trial. If standard hospital supplies are being used then temperature monitoring is not required. Do not use beyond the expiration date stamped on the vial. Drug name, dose, route, batch and expiry should all be documented in the patient's drug chart or other applicable source documents to enable source data verification of the randomized treatment and availability of all information required for Safety reporting purposes.

The investigator must agree to use the investigational product only in accordance with the protocol.

9. Adverse events and serious adverse events

The investigator is responsible for the detection and documentation of events meeting the criteria and definition of an adverse event (AE) or a serious adverse event (SAE) as provided in this protocol. During the study, when there is a safety evaluation, the investigator or site staff will be responsible for detecting AEs and SAEs, as detailed in this section of the protocol

9.1 Definition of an Adverse Event (AE)

Any untoward medical occurrence in a patient or clinical investigation subject, temporarily associated with the use of a medicinal product, whether or not considered related to the medicinal product.

An AE can therefore be any unfavourable and unintended sign (including an abnormal laboratory finding), symptom, or disease (new or exacerbated) temporally associated with the use of a medicinal product,

whether or not considered related to the medicinal product. For marketed medicinal products, this also includes failure to produce benefits (i.e. lack of efficacy), abuse or misuse.

Examples of an AE **include**:

- Exacerbation of a chronic or intermittent pre-existing condition including either an increase in frequency and/or intensity of the condition.
- New conditions detected or diagnosed after investigational product administration even though it may have been present prior to the start of the study.
- Signs, symptoms, or the clinical sequelae of a suspected interaction.
- Signs, symptoms, or the clinical sequelae of a suspected overdose of either investigational product or a concurrent medication (overdose per se should not be reported as an AE/SAE).

Examples of an AE **do not include** a/an:

- Medical or surgical procedure (e.g. endoscopy, appendectomy); the condition that leads to the procedure is an AE.
- Situations where an untoward medical occurrence did not occur (social and/or convenience admission to hospital).

In this study, AEs may include pre- or post-treatment events that occur as a result of protocol-mandated procedures (i.e. invasive procedures, modification of subjects' previous therapeutic regimen).

9.2 Definition of a Serious Adverse Event (SAE)

A serious adverse event is any untoward medical occurrence that, at any dose:

a) Results in death,

b) is life-threatening,

Note: The term 'life-threatening' in the definition of 'serious' refers to an event in which the subject was at risk of death at the time of the event. It does not refer to an event, which hypothetically might have caused death if it were more severe.

c) requires hospitalisation or prolongation of an existing hospitalisation,

Note: In general, hospitalisation signifies that the subject has been detained (usually involving at least an overnight stay) at the hospital or emergency ward for observation and/or treatment that would not have been appropriate in the physician's office or out-patient setting. Complications that occur during hospitalisation are AEs. If a complication prolongs hospitalisation or fulfils any other serious criteria, the

event is serious. When in doubt as to whether 'hospitalisation' occurred or was necessary, the AE should be considered serious.

Hospitalisation for elective treatment of a pre-existing condition that did not worsen from baseline is not considered an AE.

d) Results in disability/incapacity, or

Note: The term disability means a substantial disruption of a person's ability to conduct normal life functions. This definition is not intended to include experiences of relatively minor medical significance such as uncomplicated headache, nausea, vomiting, diarrhoea, influenza, and accidental trauma (e.g. sprained ankle) which may interfere or prevent everyday life functions, but do not constitute a substantial disruption.

e) Is a congenital abnormality / birth defect.

Medical and scientific judgement should be exercised in deciding whether reporting is appropriate in other situations, such as important medical events that may not be immediately life-threatening or result in death or hospitalisation, but may jeopardise the subject or may require medical or surgical intervention to prevent one of the other outcomes listed in the above definition. These should also be considered serious. Examples of such events are invasive or malignant cancers, intensive treatment in an emergency room or at home for allergic bronchospasm, blood dyscrasias or convulsions that do not result in hospitalisation, or development of drug dependency or abuse.

9.3 Clinical Laboratory Abnormalities and Other Abnormal Assessments as AEs and SAEs

Abnormal laboratory findings (e.g. clinical chemistry, haematology, urinalysis) or other abnormal assessments (e.g. ECG, vital signs) that are judged by the investigator as clinically significant will be recorded as AEs or SAEs if they meet the definition of an AE, as defined in Section 9.1 or SAE as defined in Section 9.2. Clinically significant abnormal laboratory findings or other abnormal assessments that are detected during the study or are present at baseline and significantly worsen following the start of the study will be reported as AEs or SAEs. However, clinically significant abnormal laboratory findings or other abnormal assessments that are associated with a disease reported in the medical history, unless judged by the investigator as more severe than expected for the subject's condition, or that are present or detected at the start of the study and do not worsen, will not be reported as AEs or SAEs.

The investigator will exercise his or her medical and scientific judgement in deciding whether an abnormal laboratory finding or other abnormal assessment is clinically significant.

9.4 Time Period, Frequency, and Method of Detecting AEs and SAEs

All adverse events will be recorded between the time of consent and discharge or Day 7 (whichever occurs first) post study drug administration. Serious adverse events will continue to be recorded until 90 days post study drug administration. Each subject will be monitored regularly by the investigator and study personnel for adverse events occurring during the first 24 hours post study drug administration. During the in-clinic treatment period, the investigator or designee will enquire about AEs by asking the following non-leading questions:

During the initial AE enquiry subjects should be asked:

"How are you feeling?"

At subsequent interviews subjects should be asked:

"Since you were last asked, have you felt unwell or different from usual?"

9.5 Recording of AEs and SAEs

When an AE/SAE occurs, it is the responsibility of the investigator to review all documentation (e.g. hospital progress notes, laboratory, and diagnostic reports) relative to the event. The investigator will then record all relevant information regarding an AE/SAE in to the CRF.

For each adverse event, start and stop dates, action taken, outcome, intensity (see Section 9.8.1) and relationship to study product (causality) (see Section 9.8.2) must be documented. If an AE changes in frequency or intensity during a study, a new entry of the event must be made in the CRF.

The investigator will attempt to establish a diagnosis of the event based on signs, symptoms, and/or other clinical information. In the absence of a diagnosis, the individual signs/symptoms should be documented.

All details of any treatments initiated due to the adverse event should be recorded in the subject's notes and the CRF.

9.6 Prompt Reporting of SAEs to the Study Management Centre

Once an investigator becomes aware that an SAE has occurred in a study subject, he/she will immediately notify the Study Management Centre (Melbourne Brain Centre, Royal Melbourne Hospital, University of Melbourne) by contacting the study monitor via telephone to notify him/her of the event. The SAE form must be completed as thoroughly as possible with all available details of the event, signed by the investigator (or appropriately qualified designee), and faxed to the study monitor within 24 hours of first becoming aware of the event.

If the investigator does not have all information regarding an SAE, ***he/she will not wait to receive additional information before notifying the study monitor*** of the event and completing the form. The form will be updated when additional information is received.

The investigator will always provide an assessment of causality at the time of the initial report as described in Section 9.8.2, "Assessment of Causality". If data obtained after reporting indicates that the assessment of causality is incorrect, then the SAE form may be appropriately amended, signed and dated, and resubmitted to the Study Management Centre.

In accordance with local IEC requirements, the investigator must also notify their Ethics Committee of any SAEs according the guidelines of the Ethics Committee.

The investigator, and others responsible for subject care, should institute any supplementary investigations of serious adverse events based on their clinical judgement of the likely causative factors. This may include seeking further opinion from a specialist in the field of the adverse event.

9.7 Expeditable Events

Expeditable events are those adverse events that are **CAUSALLY** related to the study product, **AND** that are both **SERIOUS** (see Section 9.2) and **UNEXPECTED** (see Section 9.8.3). Such events are subject to expedited reporting to regulatory authorities and will be reported within the stipulated timelines.

9.8 Evaluating AEs and SAEs

9.8.1 Assessment of Intensity

The investigator will make an assessment of intensity for each AE and SAE reported during the study. The assessment will be based on the investigator's clinical judgement. The intensity of each AE and SAE recorded in the CRF should be assigned to one of the following categories:

Mild: An event that is easily tolerated by the subject, causing minimal discomfort and not interfering with everyday activities.

Moderate: An event that is sufficiently discomforting to interfere with normal everyday activities.

Severe: An event which is incapacitating and prevents normal everyday activities.

An AE that is assessed as severe should not be confused with an SAE. Severity is a category utilised for rating the intensity of an event; and both AEs and SAEs can be assessed as severe. An event is defined as "serious" when it meets one of the pre-defined outcomes as described in Section 9.2 "Definition of an SAE".

9.8.2 Assessment of Causality

The investigator is obligated to assess the relationship between investigational product and the occurrence of each AE/SAE. The investigator will use clinical judgment to determine the relationship. Alternative causes, such as natural history of the underlying diseases, concomitant therapy, other risk factors, and the temporal relationship of the event to the investigational product will be considered and investigated. The investigator will also consult the CIB and/or product information in the determination of his/her assessment.

The causal relationship to the study product assessed by the Investigator (or medically qualified delegate) should be assessed using the following classifications:

Not Related In the Investigator's opinion, there is not a causal relationship between the study product and the adverse event.

Unlikely The temporal association between the adverse event and study product is such that the study product is not likely to have any reasonable association with the adverse event.

Possible The adverse event could have been caused by the study subject's clinical state or the study product.

Probable The adverse event follows a reasonable temporal sequence from the time of study product administration, abates upon discontinuation of the study product and cannot be reasonably explained by the known characteristics of the study subject's clinical state.

Definitely The adverse event follows a reasonable temporal sequence from the time of study product administration or reappears when study product is reintroduced.

There may be situations when an SAE has occurred and the investigator has minimal information to include in the initial report to the DSMC. However, it is very important that the investigator always makes an assessment of causality for every event prior to transmission of the SAE form the DSMC. The investigator may change his/her opinion of causality in light of follow-up information, amending the SAE form accordingly. The causality assessment is one of the criteria used when determining regulatory reporting requirements.

9.8.3 Assessment of Expectedness

Expected An adverse reaction, the nature or severity of which is consistent with the applicable product information (e.g. Investigator's Brochure for an unapproved medicinal product).

Unexpected An adverse reaction, the nature or severity of which is not consistent with information in the relevant source document (e.g. Investigator's Brochure for an unapproved medicinal product).

9.9 Follow-up of AEs and SAEs

After the initial AE/SAE report, the investigator is required to proactively follow each subject and provide further information to the DSMC on the subject's condition.

All AEs and SAEs documented at a previous visit/contact and are designated as ongoing, will be reviewed at subsequent visits/contacts.

All AEs and SAEs will be followed until resolution, until the condition stabilises, until the event is otherwise explained, or until the subject is lost to follow-up. Once resolved, the appropriate AE/SAE CRF page(s) will be updated. The investigator will ensure that follow-up includes any supplemental investigations as may be indicated to elucidate the nature and/or causality of the AE or SAE. This may include additional laboratory tests or investigations, histopathological examinations, or consultation with other health care professionals.

New or updated information will be recorded on the originally completed SAE form, with all changes signed and dated by the investigator. The updated SAE form should be resent to the Study Management Centre.

9.10 Post-study AEs and SAEs

A post-study AE/SAE is defined as any event that occurs outside the AE/SAE detection period as defined in Section 9.4 "Time Period, Frequency, and Method of Detecting AEs and SAEs" of the protocol.

Investigators are not obligated to actively seek AEs or SAEs in former study participants. However, if the investigator learns of any SAE, including death, at any time after a subject has been discharged from the study, and he/she considers the event reasonably related to the investigational product, the investigator will promptly notify the DSMC.

10. Subject completion and discontinuation

10.1 Subject Completion

Subjects will have completed their involvement with the study when the follow up visit has occurred at 90 days, data has been entered into the CRF, and any queries or inconsistencies relating to the data have been resolved with the investigator.

10.2 Subject Withdrawal

Subjects who elect to withdraw from the study should attend a final withdrawal visit. The date and reason for patient withdrawal should be recorded on the 'Study End' page of the CRF.

10.3 Discontinuation Criteria

The study may be terminated prematurely by the principal investigator or his/her designee and the sponsor. Reasons include but are not limited to:

- The number and/or severity of adverse events justify discontinuation of the study
- New data become available which raise concern about the safety of the investigational product, so that continuation might cause unacceptable risks to subjects.

In addition the Sponsor reserves the right to discontinue the trial prior to inclusion of the intended number of subjects, but intends only to exercise this right for valid scientific or administrative reasons.

After such a decision, the Investigator must contact all participating subjects within two weeks, and written notification must be sent to the Ethics Committee.

11. Case report form

A Case Report Form (CRF) will be completed for each study subject summarising all clinical screening and study data. Subjects will only be referred to in the CRF by their subject number and initials in order to retain subject confidentiality. The completed original CRFs are to be sent to the Sponsor as soon as practical after completion and review. A copy of each completed CRF is to be retained by the Investigator for a period of time as determined by local regulations. The identification of data to be recorded directly in to the CRF (i.e. no prior written or electronic record of data), and to be considered to be source data, is outlined in the Source Document Designation Form.

12. Data analysis and statistical considerations

12.1 Objective

The primary objective of the study is to test the hypothesis that the thrombolytic tenecteplase (TNK) is superior to current best practice (alteplase 0.9mg/kg or no lysis) in achieving excellent functional outcome or return to the premorbid Modified Rankin Scale in acute ischemic stroke patients presenting with an ischemic stroke due to a large vessel occlusion and who have a significant volume penumbral tissue on multimodal CT or MRI, within 24 hours after symptom onset.

12.2 Endpoints

Primary Outcome

- The proportion of patients with Modified Rankin Scale (mRS) 0-1 (no disability) or return to baseline mRS (if baseline premorbid mRS =2) at 3 months.

Secondary Outcomes

Efficacy

- Proportion of patients achieving early clinical improvement (reduction in acute – 24 hour NIHSS score of ≥8 or 24 hour NIHSS 0-1).
- Proportion of patients with Modified Rankin Scale 0-2 at 3 months
- Ordinal analysis of mRS at 3 months
- Proportion of patients with TICI 2b/3 on initial DSA run prior to thrombectomy

Safety

- Proportion of patients with symptomatic intra-cerebral haemorrhage (sICH) defined as: parenchymal haematoma type 2 (PH2) within 36 hours of treatment combined with neurological deterioration leading to an increase of ≥4 points on the NIHSS from baseline, or the lowest NIHSS value between baseline and 24 hours
- Proportion of patients with death due to any cause
- Proportion of patients with Modified Rankin Scale (mRS) 5-6 at 3 months (severe disability or death).

All secondary and safety outcomes will also be analysed adjusted for: age, NIHSS, randomisation time to groin puncture time in bins, including a bin for no EVT performed

Exploratory outcomes

- Proportion of patients achieving reperfusion (>90% and >50%) at 24 hours post stroke
- Infarct growth at 24 hours.
    - Proportion of patients achieving recanalisation at 24 hours post stroke

12.3 Sample size

An estimated total sample size of 740 patients (equally distributed between two study arms) will yield 80% power to detect an absolute difference of at least 10% in proportion of patients achieving the primary outcome between two arms using two-sided statistical significance threshold of p=0.05. The estimates in the 'current best practice' group are based on the outcomes of the EXTEND trial where the proportions of LVO patients with the primary outcome (3-month mRS 0-1) were: placebo arm 25%, alteplase arm 28%, with the more conservative assumed proportion taken as 28%. The treatment effect of tenecteplase was based upon the pooled analysis of phase II tenecteplase LVO data where the tenecteplase treatment effect size was 19% greater than alteplase. A more conservative treatment effect of 10% has been assumed for ETERNAL.

Adaptive increase[22] in sample size is planned if the result of interim analysis using data from the first 592 patients are promising, as per the methodology of Mehta and Pocock. The maximum sample size is capped at 1000 patients (500 per arm).[23].

12.4 Interim/Safety analysis

Two safety parameters (deaths and symptomatic hemorrhages within 36 hours of intervention) will be monitored by the Independent Data Safety Monitoring Committee (DSMC) after each 100 patients have been enrolled. If there are concerns about the safety of participants, DSMC will make a recommendation to the trial steering committee about continuing, stopping, or modifying the trial. To compare the safety of current best practice therapy versus IV tenecteplase, two safety parameters - mortality at three months and the incidence of symptomatic ICH within 36 hours of intervention, will be tested independently. The Haybittle-Peto procedure for generating early stopping boundaries will be used. A recommendation of early termination due to safety reasons will be considered by the DSMC if the corresponding Haybittle-Peto boundary (p=0.001, Z=3) at a given interim analysis is crossed.

No interim analyses for the primary outcome are planned apart from the analysis for the purposes of adaptive sample size re-estimation as per the “promising zone” methodology of Mehta and Pocock based on the data from the first 592 patients.

12.5 Statistical analysis

The analysis will be conducted following intention-to-treat principles. All outcomes and analyses are prospectively categorized as primary, secondary or exploratory. Differences in all endpoints between the 2 arms of the study will be tested independently at the two-tailed 0.05 level of significance. All estimates of treatment effects will be presented with 95% confidence intervals. No formal adjustments will be undertaken to constrain the overall type I error associated with the secondary and exploratory analyses. Their purpose is to supplement evidence from the primary analysis to more fully characterise the treatment effect. Results from the secondary analyses will be interpreted in this context. Descriptive statistics will be generated for each of the measures used in study.

The primary outcome will be analysed using modified Poisson regression with modified Rankin Scale (mRS) 0-1 (no disability) or return to premorbid mRS at 3 months as the dependent variable, treatment arm as an independent variable, and age, baseline NIHSS score, and randomisation-to-groin puncture time (categorized as no EVT, <2 hours, and >2 hours) as treatment covariates. Treatment effect will be presented as adjusted Risk Ratio with corresponding 95% confidence interval (95%CI).

The analyses of dichotomous secondary efficacy and safety outcomes will be conducted using modified Poisson regression with achieving the respective outcome as the dependent variable, treatment arm as an independent variable, and age, baseline NIHSS score, and randomisation-to-groin puncture time (categorized as no EVT, <2 hours, and >2 hours) as treatment covariates. Treatment effects will be presented as adjusted Risk Ratios with corresponding 95% confidence interval (95%CI).

For the ordinal analysis of the day 90 mRS, an ordinal logistic regression analysis model or an assumption-free Generalized OR model[24] analysis will be undertaken on the full range (0-6) of the scale depending on whether the proportional odds assumption is satisfied.

Exploratory outcome analyses will be carried out according to standard statistical principles for comparison of parametric or non-parametric distributions as appropriate.[25]

The details of the statistical analysis will be summarized in a separate Statistical Analysis Plan prior to the lock of the trial data.

13. Data management

Data will be recorded in a paper and electronic CRF; the electronic database will be maintained at the Melbourne Brain Centre. Access to the database will be secure and password protected, and managed by Data Managers. Access to the system will only be granted after documented training. All information collected for this study will have identifying information removed, and be kept private, confidential and secure.

14. Study monitoring

Study monitoring will be managed by the ETERNAL LVO Coordinating Centre or a suitably qualified designee for the sites outside Australia. Best conduct of the study will be ensured through frequent contacts by phone and in person with the responsible Investigator, with the purpose of facilitating the work and fulfilling the objectives of the study. Site visits will enable the Monitor to maintain current, personal knowledge of the study through review of the records, comparison with source documents, and observation and discussion of the conduct of the study with the Investigator. The Monitor is responsible for monitoring adherence to the Protocol and completion of the CRF. They are also responsible for the organisation, monitoring, supply of study materials and quality assurance of the study.

In order to ensure the accuracy of data, direct access to source documents by the representatives of both the Study Monitor and regulatory authorities is mandatory.

The trial will be managed by a Steering committee, with Co-chairs, comprising of investigators from each participating centre. An independent Data Safety Monitoring Board (DSMB) will also be convened/implemented.

Anonymity of the subject will be maintained at all times. The ETERNAL LVO Coordinating Centre reserves the right to terminate the study for refusal of the Investigator/Institution to supply source documentation of work performed in the study.

14.1 Curriculum Vitae and Other Documentation

In order to comply with regulatory requirements, all Investigators signing the Protocol and Co-investigators should provide a current, signed and dated Curriculum Vitae (CV). The CV should include name, title, occupation, education, research experience and present and former positions. A Staff Delegation/Signature List of all personnel involved in the conduct of the study is also required. To be eligible for site selection, units are required to have an established intravenous alteplase program with multimodal CT or MRI imaging as standard procedure.

14.2 Investigator Responsibility

Except where the Principal Investigator's signature is specifically required, it is understood that the term 'Investigator' as used in this Protocol and on the CRFs refers to the Principal Investigator or an appropriately qualified member of the staff that the Principal Investigator designates to perform specified duties of the Protocol. The Principal Investigator is ultimately responsible for the conduct of all aspects of the study.

14.3 Study Report

Upon completion of the study, a clinical study report will be prepared by the Principal Clinical Investigators. The data collected will remain the property of the study Steering Committee, with the database hosted by University of Melbourne. The results of the study will be submitted for publication in peer-reviewed journals.

15. Administrative Procedures

15.1 Ethical Considerations

Information on side effects of the test and reference formulations is summarised in Section 7 of this protocol. The monitoring and safety guidelines are outlined in the Monitoring Guidelines for the study. The amount of blood to be sampled in the study is not considered to be excessive in healthy adult subjects. This study will be carried out according to the Declaration of Helsinki, the NHMRC National Statement on Ethical Conduct in Research Involving Humans (1999) and the Notes for Guidance on Good Clinical Practice as adopted by the Australian Therapeutic Goods Administration (2000) (CPMP/ICH/135/95) and the ICH GCP Guidelines.

15.2 Ethical Review Committee

The Protocol will be submitted for approval to the appropriate Ethics Committee, and written approval obtained, before volunteers are recruited and subjects are enrolled. The Investigators will receive all the documentation needed for submitting the present Protocol to the Ethics Committee. A copy of the respective approval letters will be transmitted to the Study Monitor before starting the study. The

composition of the Ethics Committee will also be provided to the Study Monitor. If approval is suspended or terminated by the Ethics Committee, the Investigator will notify the Study Monitor immediately.

It is the responsibility of the Investigator to report study progress to the Ethics Committee as required or at intervals not greater than one year.

The Principal Investigator, or his/her nominee, will be responsible for reporting any serious adverse events to the Ethics Committee as soon as possible, and in accordance with the guidelines of the Ethics Committee.

15.3 Regulatory Authorities

The study will be submitted for approval by the local regulatory authorities. SAEs will be notified according to the requirements of the local regulatory authorities.

In agreeing to the provisions of the Protocol, these responsibilities are accepted by the Investigator.

15.4 Informed Consent

As treatment efficacy in acute ischemic stroke is strongly time dependent it is critical that trial procedures do not unduly delay initiation of standard therapy. Existing data on tenecteplase indicate that it is very unlikely to be less effective or higher risk than alteplase. To minimize delay in "door-to-needle" we will request HREC approval to use the section 42A clause in the Victorian Guardianship and Administration Act that emergency treatment that is necessary to prevent serious injury to the patient's health may be commenced without consent and the analogous provisions in the updated Victorian Medical Treatment Planning and Decisions Act 2016. We will then undertake standard written consent to continue follow-up in the trial. For jurisdictions that do not permit this approach we will use an abbreviated 1-page patient information form as used in the TASTE randomized trial of tenecteplase vs alteplase (ACTRN12613000243718). Each participant (or authorised representative where this is applicable) will be given a full explanation of the nature and purposes of the study, and a copy of the Patient Information Sheet to review. Once the essential study information has been provided, and the Investigator is assured that each patient or their representative understands the implications of participating or continuing in the study as appropriate, the subjects or their representatives will be asked to give consent to the study by signing the informed consent form. The consent forms shall be signed and dated by the appropriate parties.

A notation of the nature of consent will be made on the subject's medical history/patient notes, and recorded in the eCRF. The completed consent forms will be retained by the Investigator and a copy of these will be provided by the Investigator to the subject.

15.5 Subject Reimbursement

No subject reimbursement is provided for participation in this trial.

15.6 Emergency Contact with Investigators

All subjects will be provided with contact details of whom to contact in the case of an emergency.

15.7 Investigator Indemnification

The study is being conducted subject to the 'Guidelines for Compensation for Injury Resulting from Participation in a Company-sponsored Clinical Trial' published by the Medicines Australia. Company Name will reimburse subjects for costs of medical care that occur as a result of complications directly related to participation in this study.

15.8 Financial Aspects

The conduct of the study is subject to a Financial Agreement between Company Name and the Investigator or Institution.

15.10 Protocol Amendments

No changes (amendments) to the Protocol may be implemented without prior approval from the appropriate Ethics Committee. In the case of substantial amendment, approval from the Competent Regulatory Authority will be sought before implementation. If a Protocol amendment requires changes to the Informed Consent Form, the revised Informed Consent Form, prepared by the Investigator, must be approved by the Ethics Committee.

It is the responsibility of the Investigator to submit the amendment to the Ethics Committee and Competent Regulatory Authority for their approval. Completed and signed Protocol amendments will be circulated to all those who were on the circulation list for the original Protocol.

The original signed copy of amendments will be kept in the Study File with the original Protocol. It should be noted that where an amendment to the Protocol substantially alters the study design or the potential risks to the subjects, each subject's consent to continue participation should be obtained.

15.11 Protocol Compliance

The instructions and procedures specified in this Protocol require diligent attention to their execution. Should there be questions or consideration of deviation from the Protocol, clarification will be sought from the Study Monitor. Any subject treated in a manner that deviates from the Protocol, or who is admitted into the study but is not qualified according to the Protocol as amended by the Investigator, may be ineligible for analysis and thereby compromise the study.

Only when an emergency occurs that requires a departure from the Protocol for an individual will there be such a departure. The nature and reasons for the Protocol violation shall be recorded in the CRF.

The Investigator and designees will comply with all applicable federal, state and local laws.

15.12 Archives: Retention of Study Records

All source documents, CRFs and trial documentation will be kept by the Investigator for the appropriate retention period as stipulated by local regulations and ICH-GCP[1].

16 References

---

[1] Feigin VL, Forouzanfar MH, Krishnamurthi R, Mensah GA, Connor M, Bennett DA, Moran AE, Sacco RL, Anderson L, Truelsen T, O'Donnell M, Venketasubramanian N, Barker-Collo S, Lawes CM, Wang W, Shinohara Y, Witt E, Ezzati M, Naghavi M, Murray C; Global Burden of Diseases, Injuries, and Risk Factors Study 2010 (GBD 2010) and the GBD Stroke Experts Group. Global and regional burden of stroke during 1990-2010: findings from the Global Burden of Disease Study 2010. Lancet. 2014 18;383(9913):245-54.

[2] 2017 Stroke Foundation Audit (https://strokefoundation.org.au/Events/2016/12/02/Acute%20Services%20Audit%202017%20announcement)

[3] Hacke W, Donnan G, Fieschi C, Kaste M, von Kummer R, Broderick JP, et al. Association of outcome with early stroke treatment: Pooled analysis of atlantis, ecass, and ninds rt-pa stroke trials. *Lancet*. 2004;363:768-774

[4] Hacke W, Kaste M, Fieschi C, von Kummer R, Davalos A, Meier D, et al. Randomised double-blind placebo-controlled trial of thrombolytic therapy with intravenous alteplase in acute ischaemic stroke (ecass ii). Second european-australasian acute stroke study investigators. *Lancet*. 1998;352:1245-1251

[5] Berkhemer OA, Fransen PSS, Beumer D, van den Berg LA, Lingsma HF, Yoo AJ, et al. A randomized trial of intraarterial treatment for acute ischemic stroke. *New England Journal of Medicine*. 2015;372:11-20

[6] Campbell BCV, Mitchell PJ, Kleinig TJ, Dewey HM, Churilov L, Yassi N, et al. Endovascular therapy for ischemic stroke with perfusion-imaging selection. *New England Journal of Medicine*. 2015;372:1009-1018

[7] Goyal M, Demchuk AM, Menon BK, Eesa M, Rempel JL, Thornton J, et al. Randomized assessment of rapid endovascular treatment of ischemic stroke. *New England Journal of Medicine*. 2015;372:1019-1030

[8] Campbell BCV, Mitchell PJ, Churilov L, Yassi N, Kleinig TJ, Dowling RJ, Yan B, Bush SJ, Dewey HM, Thijs V, Scroop R, Simpson M, Brooks M, Asadi H, Wu TY, Shah DG, Wijeratne T, Ang T, Miteff F, Levi CR, Rodrigues E, Zhao H, Salvaris P, Garcia-Esperon C, Bailey P, Rice H, de Villiers L, Brown H, Redmond K, Leggett D, Fink JN, Collecutt W, Wong AA, Muller C, Coulthard A, Mitchell K, Clouston J, Mahady K, Field D, Ma H, Phan TG, Chong W, Chandra RV, Slater LA, Krause M, Harrington TJ, Faulder KC, Steinfort BS, Bladin CF, Sharma G, Desmond PM, Parsons MW, Donnan GA, Davis SM; EXTEND-IA TNK Investigators. Tenecteplase versus Alteplase before Thrombectomy for Ischemic Stroke. N Engl J Med. 2018 Apr 26;378(17):1573-1582

[9] Nogueira RG, Jadhav AP, Haussen DC, et al.N Engl J Med. Thrombectomy 6 to 24 Hours after Stroke with a Mismatch between Deficit and Infarct. N Engl J Med 2018 4;378:11-21.

[10] Albers G, Marks M, Kemp S, et al. Thrombectomy for Stroke at 6 to 16 Hours with Selection by Perfusion Imaging. N Engl J Med 2018 4;378:11-21.

[11] Bivard A, Levi C, Krishnamurthy V, et al. Perfusion computed tomography to assist decision making for stroke thrombolysis. *Brain.* 2015;138(Pt 7):1919-31.

[12] Ma, H, Campbell BCV, Mitchell PJ, Churilov L, Yassi N, Kleinig TJ, Dowling RJ, Yan B, Bush SJ, Dewey HM, Thijs V, Scroop R, Simpson M, Brooks M, Asadi H, Wu TY, Shah DG, Wijeratne T, Ang T, Miteff F, Levi CR, Rodrigues E, Zhao H, Salvaris P, Garcia-Esperon C, Bailey P, Rice H, de Villiers L, Brown H, Redmond K, Leggett D, Fink JN, Collecutt W, Wong AA, Muller C, Coulthard A, Mitchell K, Clouston J, Mahady K, Field D, Ma H, Phan TG, Chong W, Chandra RV, Slater LA, Krause M, Harrington TJ, Faulder KC, Steinfort BS, Bladin

CF, Sharma G, Desmond PM, Parsons MW, Donnan GA, Davis SM; EXTEND InvestigatorsThrombolysis for Stroke Guided by Perfusion Imaging up to 9 Hours after Onset. NEJM 18-13046

[13] Parsons M, Spratt N, Bivard A, et al. A Randomized Trial of Tenecteplase versus Alteplase for Acute Ischemic Stroke. N Engl J Med. 2012;366:1099- 107.
[14] Kate M, Wannamaker R, Kamble H, Riaz P, Gioia LC, Buck B, Jeerakathil T, Smyth P, Shuaib A, Emery D, Butcher K. Penumbral Imaging-Based Thrombolysis with Tenecteplase Is Feasible up to 24 Hours after Symptom Onset. J Stroke. 2018 Sep;20(3):415.

[15] De Werf F, Adgey J, Ardissino D, Armstrong PW, et al. Single-bolus tenecteplase compared with front-loaded alteplase in acute myocardial infarction: The assent-2 double-blind randomised trial. *Lancet*. 1999;354:716-722

[16] Logallo N, Novotny V, Assmus J, Kvistad CE, Alteheld L, Rønning OM, Thommessen B, Amthor KF, Ihle-Hansen H, Kurz M, Tobro H, Kaur K, Stankiewicz M, Carlsson M, Morsund Å, Idicula T, Aamodt AH, Lund C, Næss H, Waje-Andreassen U, Thomassen L. Tenecteplase versus alteplase for management of acute ischaemic stroke (NOR-TEST): a phase 3, randomised, open-label, blinded endpoint trial. Lancet Neurol. 2017 16:781-788.

[17] Parsons MW, Miteff F, Bateman GA, Spratt N, Loiselle A, Attia J, et al. Acute ischemic stroke: Imaging-guided tenecteplase treatment in an extended time window. *Neurology*. 2009;72:915-921

[18]Campbell BCV, Mitchell PJ, Kleinig TJ, Dewey HM, Churilov L, Yassi N, et al. Endovascular therapy for ischemic stroke with perfusion-imaging selection. *New England Journal of Medicine*. 2015;372:1009-1018
[19] Garcia-Esperon C, Bivard A, Levi C, Parsons M. Use of computed tomography perfusion for acute stroke in routine clinical practice: Complex scenarios, mimics, and artifacts. Int J Stroke. 2018;13(5):469-472.

[20] Bivard A, Huang X, McElduff P, Levi CR, Campbell BC, Cheripelli BK, Kalladka D, Moreton FC, Ford I, Bladin CF, Davis SM, Donnan GA, Muir KW, Parsons MW. Impact of Computed Tomography Perfusion Imaging on the Response to Tenecteplase in Ischemic Stroke: Analysis of 2 Randomized Controlled Trials. Circulation. 2017 Jan 31;135(5):440-448.
[21] Bladin CF, Cadilhac DA. Effect of telestroke on emergent stroke care and stroke outcomes. **Stroke** 2014; 45(6):1876-80.

[22] Mehta CR, Pocock SJ. Adaptive increase in sample size when interim results are promising: A practical guide with examples. *Stat Med*. 2011;30:3267-3284

[23] Bivard A, Huang X, McElduff P, Levi CR, Campbell BC, Cheripelli BK, et al. Impact of computed tomography perfusion imaging on the response to tenecteplase in ischemic stroke: Analysis of 2 randomized controlled trials. *Circulation*. 2017;135:440-448

[24] Churilov L, Arnup S, Johns H, Leung T, Roberts S, Campbell BC, Davis SM, Donnan GA. An improved method for simple, assumption-free ordinal analysis of the modified Rankin scale using generalized odds ratios. Int J Stroke 2014; 9: 999–1005.

[25] David Moher,Sally Hopewell, Kenneth F Schulz, Victor Montori, Peter C Gøtzsche, P J Devereaux, Diana Elbourne, Matthias Egger, Douglas G Altman. CONSORT 2010 Explanation and Elaboration: updated guidelines for reporting parallel group randomised trials *BMJ* 2010;340:c869 doi: 10.1136/bmj.c869

17 Appendix 1 Study Schedule of Events Table

Procedure/ Investigation	Detail	Screening	Surgical Intervention Day 0 Treatment	Post Treatment		
				Day 1 (24h ±6h)	Day 90 ± 7 days	Day 360 ± 30 days
Imaging	CT Brain or MRI	x		X		
Inclusion/ exclusion criteria		x				
Informed consent		x				
Randomisation	Tenecteplase + thrombectomy vs SOC		X			
Medical History		x				
Concomitant medications		x		X		
ECG		x				
Adverse Events	Day 90 mortality or SAE only			X	X	
Routine Laboratory assessments	Haematology –Full blood examination	x				
	Clinical Chemistry	x				
	Coagulation profile	x				
Clinical examinations	mRS (D90, D360 by phone)	x			X	X
	NIHSS (24 h blinded accessor)	x		X		
	EQ5D, PROMIS10 (by phone, central blinded accessor)				X	X
